# Human immune deficiency virus‐related structural alterations in the brain are dependent on age

**DOI:** 10.1002/hbm.25423

**Published:** 2021-03-23

**Authors:** Jing Zhao, Zhe Ma, Feng Chen, Li Li, Meiji Ren, Aixin Li, Bin Jing, Hongjun Li

**Affiliations:** ^1^ School of Biological Science and Medical Engineering Beihang University Beijing China; ^2^ Department of Radiology Beijing Youan Hospital, Capital Medical University Beijing China; ^3^ Department of Radiology Henan Cancer Hospital, The Affiliated Cancer Hospital of Zhengzhou University Zhengzhou Henan China; ^4^ School of Biomedical Engineering Capital Medical University Beijing China; ^5^ Center for Infectious Diseases Beijing Youan Hospital, Capital Medical University Beijing China

**Keywords:** age effect, cART, HIV, machine learning, structural MRI

## Abstract

Currently, it is still unknown whether human immune deficiency virus (HIV)‐related structural alterations in the brain are dependent on age. With people living with HIV at different ages, we aim to investigate age‐specific structural alterations in HIV patients. Eighty‐three male HIV patients and eighty‐three age‐matched male controls were enrolled, and high‐resolution T1 weighted images were collected and analyzed with four morphological metrics. Then, statistical analyses were respectively conducted to ascertain HIV effects, age effects, and medication effects in brain structure of HIV patients, and the relationship with neuropsychological evaluations were further explored. Finally, discriminative performances of these structural abnormalities were quantitatively testified with three machine learning models. Compared with healthy controls, HIV patients displayed lower gray matter volumes (GMV), lower gyrification index, deeper sulcus depth, and larger cortical thickness (CTH). Age‐specific differences were found in GMV and CTH: young‐aged HIV patients displayed more obvious morphological alterations than middle‐aged HIV patients when comparing corresponding age‐matched healthy controls. Furthermore, age‐specific long‐term medication effect of combination antiretroviral therapy were also presented. Additionally, several subcortical structural changes were negatively associated with language, attention and motor functions. Finally, three machine learning models demonstrated young‐aged HIV patients were easier to be recognized than middle‐aged HIV patients. Our study indicated young‐aged HIV patients were more vulnerable to HIV infection in brain structure than middle‐aged patients, and future studies should not ignore the age effect in studying the HIV‐related abnormalities.

## Graphical abstract

In this study, we firstly reported that the young adult human immune deficiency virus (HIV) patients suffered more severe structural damages than middle‐aged HIV patients, but were more beneficial in the long‐term combination antiretroviral therapy medication than middle‐aged HIV patients. Taken together, HIV‐related structural alterations are dependent on age, and future studies should control the age information in order to ascertain the HIV‐specific alterations.

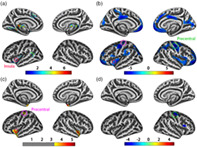

## 1 INTRODUCTION

With the successful treatment of combination antiretroviral therapy (cART), human immune deficiency virus (HIV) associated neurocognitive disorders has become the major concern for HIV infected patients (Heaton et al., 2010) and caused a series of neurocognitive deficits such as memory, language impairments that seriously affect the quality of daily living for HIV patients (Saylor et al., 2016). Several studies have reported people living with HIV are at high risk of age‐related comorbidities (Kooij et al., 2016; Schouten et al., 2014) and displayed accelerated brain aging (Kim‐Chang et al., 2019; Kuhn et al., 2018) even in viral suppression, which indicated the influences of HIV on the aging process in human brain (Chu et al., 2018; Hakkers et al., 2017).

Currently, the age of new HIV infection patients becomes younger, and the majority is between 20 and 50 years old in China with middle‐aged patients (between 30 and 40 years old) showing the highest prevalence (Qiao et al., 2019). Meanwhile, the young‐aged patients who are acquired HIV through homosexual sex increase largely in recent years, accounting for 21% of new HIV infection in United States (Johnson et al., 2014). Compared with the middle‐aged patients, young‐aged HIV patients are more vulnerable to stroke (Benjamin et al., 2016) and cognition impairments (Hosek & Zimet, 2010), indicating there may be unique brain changes in young‐aged HIV patients. Moreover, even during the adulthood, the human brain does not keep unchangeable and has to adapt to complex circumstances like learning, experience, and cognitive reserve (Lovden, Wenger, Martensson, Lindenberger, & Backman, 2013). In this condition, we hypothesized that age‐specific brain alterations may exist in HIV patients especially for young‐aged and middle‐aged HIV patients.

Structural magnetic resonance imaging (sMRI) could be quantified by plenty of brain structural metrics such as volume and cortical thickness (CTH), which have been widely used in HIV diseases (Correa et al., 2016; MacDuffie et al., 2018; Sanford et al., 2017; Sanford, Ances, et al., 2018). For example, gray matter volumetric alternations in putamen and caudate were observed in earliest HIV‐infected state (Kallianpur et al., 2020) and in primary HIV infection durations (Ragin et al., 2012), and played a key role in the multimodal classification for HIV patients (Sui et al., 2020). In addition, stable cART was reported to alleviate the volume reduction in chronically HIV infected patients with longitudinal and cross‐sectional observations (Cole et al., 2018; Sanford, Ances, et al., 2018; Sanford, Fellows, Ances, & Collins, 2018). Although many achievements have been obtained in HIV patients with sMRI, few studies have been specially conducted for the age‐dependent HIV structural alterations from young‐aged to middle‐aged adulthood, and it is also unknown whether multiple morphological metrics may gain consistent findings.

To clarify these issues, we recruited young‐ and middle‐aged HIV infected individuals and age‐matched HIV uninfected controls, and combined multiple structural metrics including gray matter volume (GMV), CTH, gyrification index (GI), and sulcus depth (SD) to comprehensively investigate the age‐dependent brain alterations for HIV patients. In addition, the relationship between structural alterations and neurocognitive test scores were explored, and the classification models with these structural alterations were also constructed with three machine learning algorithms. We speculated HIV infected individuals would display age‐dependent structural changes in the brain with four structural metrics, and the age‐dependent structural changes would better discriminate HIV infected patients from healthy controls. Moreover, since age‐dependent structural changes exist in HIV patients, age‐dependent long‐term medication effects in brain morphology may also exist.

## 2 MATERIALS AND METHODS

### 2.1 Participants

Totally, 83 patients with HIV infection and 83 demographically matched healthy control (HC) were recruited from the Department of Radiology, Beijing Youan Hospital. All subjects were male with age between 20 and 50 years. All HIV patients were infected through sexual transmission. Participants were excluded from the study if they had a history of neurological diseases or opportunistic central nervous system infection, traumatic brain injury, substance or alcohol abuse, depression, hepatitis B, or C coinfection. Demographic and clinical information on gender, age, CD4+ T cell count, ratio of CD4 and CD8+ T cell, duration of diagnosis and duration of cART were collected in HIV infected patients and summarized in Table 1. There was no significant difference in age (*p* = .403) but significant difference was shown in total intracranial volume (TIV) (*p* = .005) between HIV and HC groups. The correlations between demographic information of HIV patients and age were shown in Table S1. In order to ascertain the age‐dependent structural alterations, the subjects were further divided into two typical age subgroups: young‐aged (20–30 years old) and middle‐aged (31–50 years old) groups. Specifically, there were no significant differences between young‐aged and middle‐aged HIV groups in TIV, CD4+ T cell count, CD4/CD8 ratio and months since diagnosis except medication duration (*p* = .008). This study was approved by the Research Ethics Review Board of Beijing Youan Hospital in accordance with the declaration of Helsinki, and all volunteers provided informed consents.

**TABLE 1:** The demographical information of all subjects

	HIV	HC
Number of subjects	83	83
Age (years)	31.19 (6.66)	30.23 (8.07)
Age (young/middle)	44/39	47/36
TIV (mm^3^)^a^	1,569.26 (120.64)	1,515.77 (121.44)
CD4 (counts/cells)	504.66 (203.22)	
CD4/CD8 ratio	0.65 (0.41)	
Months since diagnosis	34.7 (34.6)	
Months since medication	26 (22)	

*Note*: Young, age between 20 and 30 years; middle, age between 31 and 50 years. Data were expressed as the mean (standard deviation).

^a^ Significant difference between groups.

Abbreviations: HC, healthy control; HIV, human immune deficiency virus; TIV, total intracranial volume.

Moreover, medication is another factor that may influence the brain structure in HIV patients, thus the subjects were further divided into two subgroups by medication duration: short medication duration (<19 months, which is the median of all medication duration) and long medication duration (>19 months). In total 83 HIV subjects, 17 subjects had no history of taking drugs, and 3 subjects took medication less than 10 days, who were excluded for further analysis. Sixty‐three patients with HIV infection were finally enrolled, and underwent the cART consisting of tenofovir/lamivudine/efavirenz or tenofovir/lamivudine/lopinavir/ritonavir, which resulted in the same CNS penetration effectiveness score for each subject (Eggers et al., 2017; Schmidt et al., 2021). Table 2 listed the detailed information of subjects in each subgroup.

**TABLE 2:** Demographical and neuropsychological information of medicated HIV patients

	Medicated HIV patients (*N* = 63)			
	Young age group		Middle age group	
Medication duration	Short‐term	Long‐term	Short‐term	Long‐term
Number of subjects	18	12	14	19
Age (years)	27.4 (2.2)	26.6 (2.3)	36.6 (4.9)	37.7 (5.2)
CD4 (counts/cells)	487.4 (160.3)	454.2 (250.5)	480.0 (200.7)	492.8 (189.0)
CD4/CD8 ratio	0.66 (0.54)	0.71 (0.46)	0.70 (0.49)	0.55 (0.28)
Months since diagnosis	9.8 (7.47)	31.73 (10.97)	18.25 (9.4)	54.50 (34.62)
Months since medication	8.8 (6.2)	31.33 (12.0)	14.6 (5.2)	47.2 (26.2)
Cognitive domain				
Language fluency	44.9 (10.5)	45.0 (6.1)	46.9 (8.3)	48.8 (11.1)
Attention	38.4 (7.5)	41.3 (5.8)	43.6 (6.5)	44.6 (5.8)
Executive function	56.1 (9.2)	55.9 (11.7)	53.9 (8.0)	57.3 (8.8)
Memory	42.5 (8.0)	40.8 (6.6)	42.0 (6.8)	47.2 (8.4)
Speed of information processing	42.2 (8.8)	44.4 (7.1)	44.9 (7.6)	47.4 (8.4)
Motor function	46.1 (9.4)	42.6 (7.4)	43.8 (11.2)	48.7 (12.0)

*Note*: Short‐term, medication duration less than 19 months; long‐term, medication duration longer than 19 months. Data were expressed as the mean (*SD*).

Abbreviation: HIV, human immune deficiency virus.

### 2.2 Neuropsychological tests

In order to comprehensively assess the cognitive abilities of the HIV subjects, six cognitive domains were evaluated: Language fluency (Animal Verbal Fluency Test), Attention (Continuous Performance Test‐Identical Pair; Wechsler Memory Scale; Paced Auditory Serial Addition Test), Executive function (Wisconsin Card Sorting Tests), Memory (Hopking Verbal Learning Test; Brief Visuospatial Memory Test), Speed of information processing (Trail Marking Test A), and Motor function (Grooved Pegboard test, dominant and nondominant) (Table 2). The differences in the cognitive domain between young‐aged and middle‐aged HIV patients are listed in Table S2.

### 2.3 Structural MRI acquisition

All participants took MRI scanning using a 3 T whole‐body scanner (Siemens, Erlangen, Germany) with a 32‐channel head coil at Beijing Youan Hospital. The imaging protocol included T1‐weighted three‐dimensional magnetization prepared rapid acquisition gradient echo sequence (repetition time/echo time/inversion time = 1,900/2.52/900 ms; voxel size = 1.0 × 1.0 × 1.0 mm^3^; flip angle = 9°). All imaging data were obtained after neuropsychological testing.

### 2.4 Structural image processing

The morphological analyses of structural MRI data were performed with the Computational Anatomy Toolbox (http://www.neuro.uni-jena.de/cat/). Briefly, the structural MRI data was firstly skull stripped and corrected for bias‐field inhomogeneity. For voxel‐wise GMV measurement, images were then segmented and normalized to the MNI standard space by DARTEL algorithm. Modulation was applied to preserve the volume of GM and all GM images were smoothed with 4‐mm FWHM Gaussian kernel. For vertex‐wise morphological indexes, the cortical surface was then reconstructed as the tessellation with thousands of triangles. Subsequently, surface inflation and registration to spherical atlas were performed, and the reconstructed surfaces were corrected for topological defects. Finally, three morphological features were computed at the vertex level, including CTH, GI, and SD. CTH represents the closest distance between the white and pial surfaces at each vertex on the tessellated surface. GI and SD are morphological indexes to assess the complexity of cortical surface: GI is defined as absolute mean curvature at each vertex, while SD measures the Euclidean distance between the central surface and its convex hull.

### 2.5 Statistical analysis

All statistical analyses were conducted using Statistical Parametric Mapping, Version 12 (Ashburner et al., 2018). First of all, the global mean of each morphological metric for whole cortical area were compared between HIV and HC groups. After that, three different levels of voxel/vertex‐wise statistical analyses were conducted consecutively. (a) HIV effects: the HIV structural abnormalities were detected using the four structural MRI metrics on the whole dataset (without age subgroups) through independent two‐samples *t* test. (b) Age effects: the whole dataset was further divided into two age subgroups (young vs. middle age), and the differences between HIV and HC were again compared with the above‐mentioned statistical process in each age subgroup. (c) Medication effects: in order to further control the influences of medication on brain structure, two age subgroups were further grouped by the medication duration (long‐term vs. short‐term), and the structural abnormalities in HIV were examined in each age subgroups with equivalent medication duration. All the statistical analyses were corrected using cluster‐level family wise error (FWE) with a threshold of 0.05 and peak‐level correction with a threshold of 0.001. Notably, in the statistical analyses of HIV effects and Medication effects, age was used as a covariate to regress out age influences, and TIV was only taken as a covariate to correct for individual head size differences in the GMV comparison.

Finally, the correlation analysis between six cognitive scores and structural abnormalities from four morphological metrics were respectively calculated in all HIV patients. The data distribution was evaluated using Kolmogorov–Smirnov test, and each cognitive score did not conform to the standard normal distribution. Therefore, nonparametric Spearman correlation was used to calculate the correlation coefficients between morphological features and cognitive scores, and *p* < .05 was thought as a significance level in the correlation analysis.

### 2.6 Classification model construction

In order to quantitatively compare the discriminative performances of different morphological features, support vector machine (SVM) was adopted to construct the classification model for HIV patients. In addition, random forest (RF) and extreme learning machine (ELM) were also used to verify the SVM results. All models employed 10‐fold cross validation to evaluate the classification performance. SVM was carried out by LibSVM (https://www.csie.ntu.edu.tw/~cjlin/libsvm/) with radial‐based function as basis kernel, and recursive feature elimination was used to determine an optimal feature subset with the most discriminative performance for classification. RF is an ensemble of numerous decision tree classifiers that generated through randomized feature subset sampling and bagging, and was conducted by Random Forests package (https://cran.r-project.org/web/packages/randomForest/). ELM is a type of feedforward neural network proposed by Guang‐Bin Huang and Siew (2006), which could provide good generalization performance with extremely fast learning speed (Zhang, Liu, Chao, Zhang, & Zhang, 2018), and it was implemented with the code (https://personal.ntu.edu.sg/egbhuang/elm_codes.html).

## 3 RESULTS

### 3.1 HIV abnormalities on whole dataset for each structural metric

**FIGURE 1:**
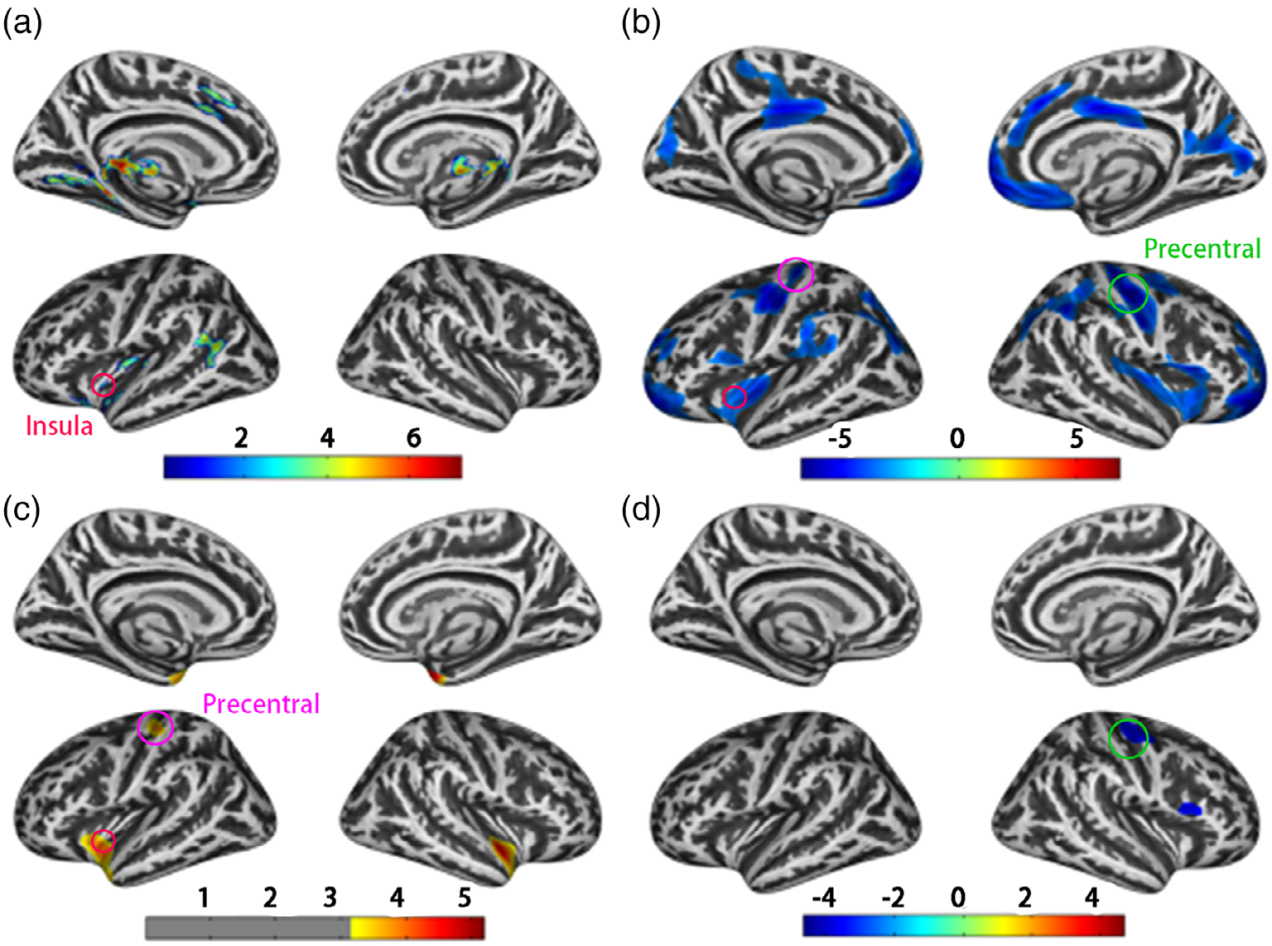
T‐value maps exhibiting significant between‐group differences (healthy control [HC] > human immune deficiency virus [HIV]) on whole dataset for each structural metric (a, gray matter volumes [GMV]; b, cortical thickness [CTH]; c, gyrification index [GI]; d, sulcus depth [SD]). Consensus brain regions among different metrics are marked with circles, and circles with same color represent the consistent abnormalities with different metrics

Differences between HC and HIV patients for each metric were firstly detected with the whole dataset. At the global mean level, there was no significant between‐group difference in GMV, while significant differences were observed in CTH, GI, and SD between HIV and HC (Figure S1). At the voxel/vertex level (Figure 1), GMV reduction was observed in HIV at bilateral thalamus, hippocampus, superior temporal regions, left inferior and middle temporal gyrus, fusiform, parahippocampus, lingual gyrus, putamen, pallidum, and right insula. HIV patients also displayed significantly larger CTH at bilateral frontal, parietal regions, precentral gyrus, postcentral gyrus, posterior cingulate cortex, supramarginal gyrus, insula, left cuneus, and right precuneus. Moreover, lower GI at the bilateral superior temporal gyrus, insula and left precentral gyrus, postcentral gyrus, and deeper SD at right precentral gyrus and pars opercularis were found in HIV patients. Notably, there were several consistent abnormalities (“circle” label in Figure 1) among four structural metrics between HIV and HC groups: GMV, CTH, and GI detected consensus region in left insula, while CTH and GI reported common abnormality in left precentral gyrus and CTH and SD in right precentral gyrus. All abnormal regions were reported based on Desikan–Killiany atlas in the study (Desikan et al., 2006).

### 3.2 Age‐dependent structural alterations for each structural metric

**FIGURE 2:**
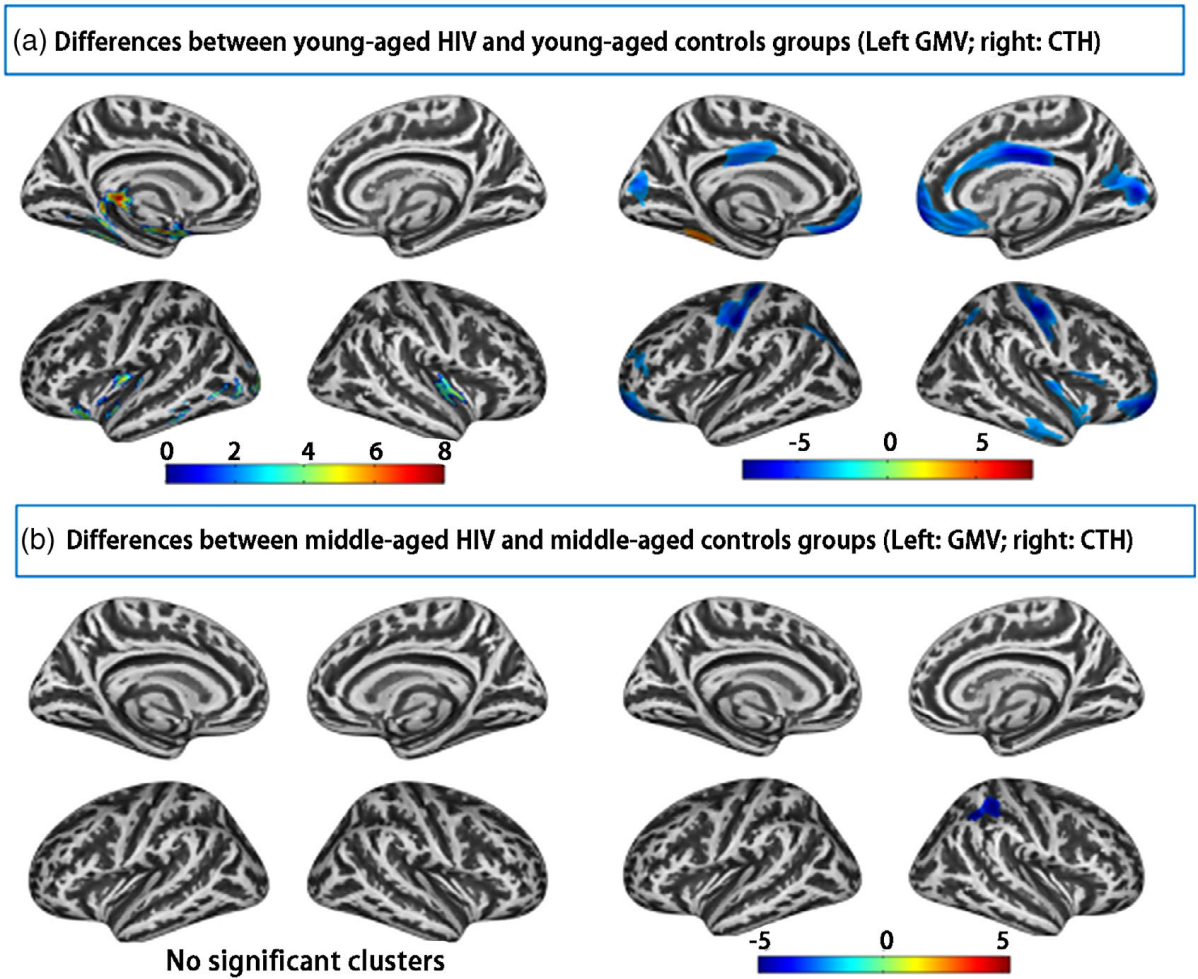
Significant differences (healthy control [HC] > human immune deficiency virus [HIV]) on gray matter volumes [GMV] and cortical thickness [CTH] between HIV patients and HC at different age subgroups

First, a full factorial design was carried out to investigate the interaction between HIV diagnosis and age on brain structure. For each structural feature, significant interaction effect between HIV and age was found (*p* < .05, FWE correction). Subsequently, the age‐dependent effects for each structural metric were discovered through further inter‐group comparisons. The GMV and CTH differences between HIV patients and HC at different age subgroups are displayed in Figure 2, and the GMV and CTH differences between young‐aged and middle‐aged HIV/HC subjects are illustrated in Figure S2. The detailed brain locations for the above‐mentioned statistical comparison are summarized in Tables S3–S6. The corresponding GI and SD differences are listed in the supplementary Figures S3 and S4. Notably, the differences between HIV and HC in young‐aged group were more severe than that in middle‐aged group, demonstrating age‐dependent HIV structural alterations in brain. In addition, HC suffered obvious structural alterations than HIV patients from young‐aged to middle‐aged adulthood (Figure S2).

### 3.3 Long‐term medication effects for each structural metric

In this respect, HIV patients were further divided into long‐term and short‐term medication duration subgroups in each age subgroup, generating a total of four subgroups (Table 2). The detailed between‐group brain regions are summarized in Table S7. Among all structural metrics, there were only significant differences in GMV. In young‐aged HIV patients, long‐term medication duration improved brain atrophy to some extent, but in middle‐aged HIV patients, there was no significant improvement.

### 3.4 Relationship with cognitive scores

**FIGURE 3:**
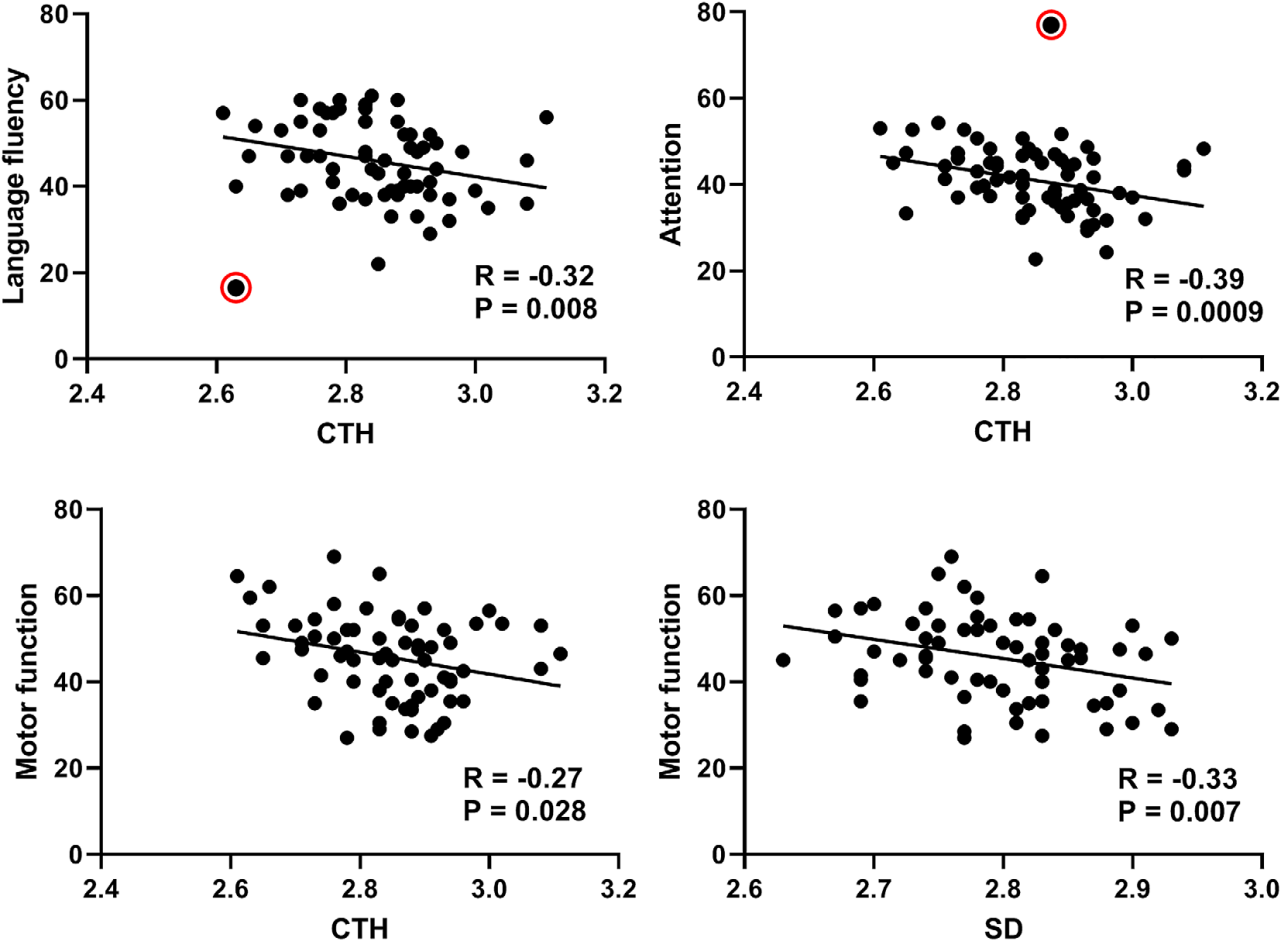
Correlation between cognitive scores and different morphological features (outliers are marked in red circles)

The Language fluency, attention and motor function scores were negatively correlated with global mean CTH (*r* = −.26/−.38/−.27, *p* < .05). The motor function score was negatively correlated with global mean *SD* (*r* = −.30, *p* < .05). Notably, there were outliers in the scatter plot of language fluency and attention scores. After excluding outliers, the correlation coefficients between language fluency/attention scores and CTH were −0.32 and −0.39, respectively (*p* < .01, Figure 3).

### 3.5 Overall classifiers performance

In this study, three classification tasks for HIV diagnosis were conducted as follows:

1. C1: all HIV versus all HC.
2. C2: young‐aged HIV versus young‐aged HC.
3. C3: middle‐aged HIV versus middle‐aged HC.

**FIGURE 4:**
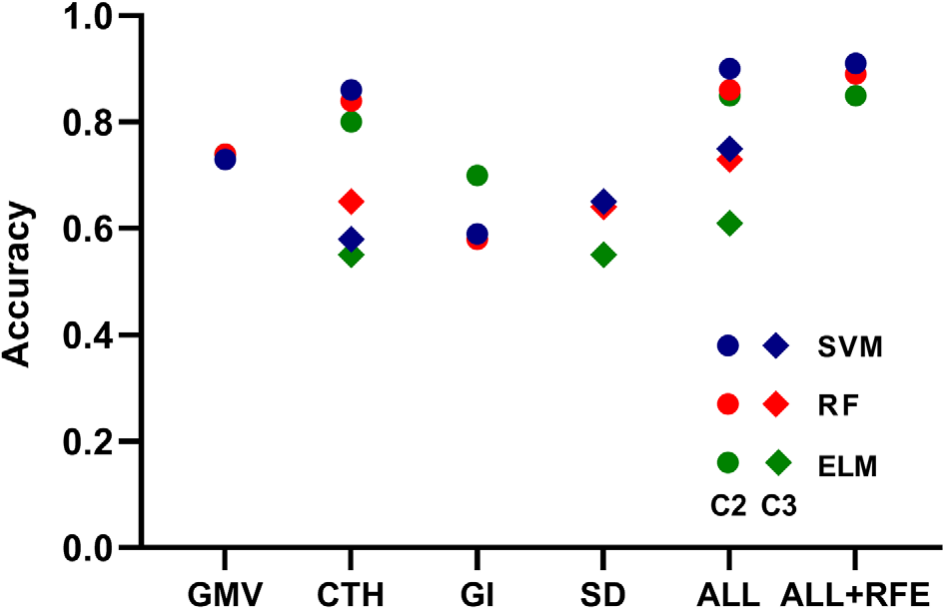
Classification performance comparison of three classifiers in two identification tasks (C2: young‐aged human immune deficiency virus [HIV] vs. young‐aged healthy control [HC]; C3: middle‐aged HIV vs. middle‐aged HC)

The classification accuracy, as well as sensitivity and specificity for each classifier are listed in Table S8. GMV and CTH outperformed GI and SD in HIV identification, and combining the multiple morphological metrics together resulted in a better identification model. Among the three classifiers, SVM obtained the best classification performance, which was followed by RF and ELM (Figure 4). Notably, young‐aged HIV patients were more precisely identified than middle‐aged HIV patients, also verifying the age‐dependent structural abnormalities in HIV patients.

## 4 DISCUSSION

In this study, the major findings were that age played an interactive role with HIV status on brain structure and young‐aged patients were more vulnerable to HIV infection. Several machine learning models also demonstrated that the discriminative performances for young‐aged HIV patients were largely better than middle‐aged HIV patients. In addition, age‐dependent medication treatment effect on brain structure was also found in young‐aged and middle‐aged HIV patients. Four structural metrics discovered widespread brain abnormalities in HIV patients, which mainly located at thalamus, hippocampus, putamen, precentral gyrus, and postcentral gyrus. Furthermore, the HIV‐related structural changes were associated with cognitive functions.

**FIGURE 5:**
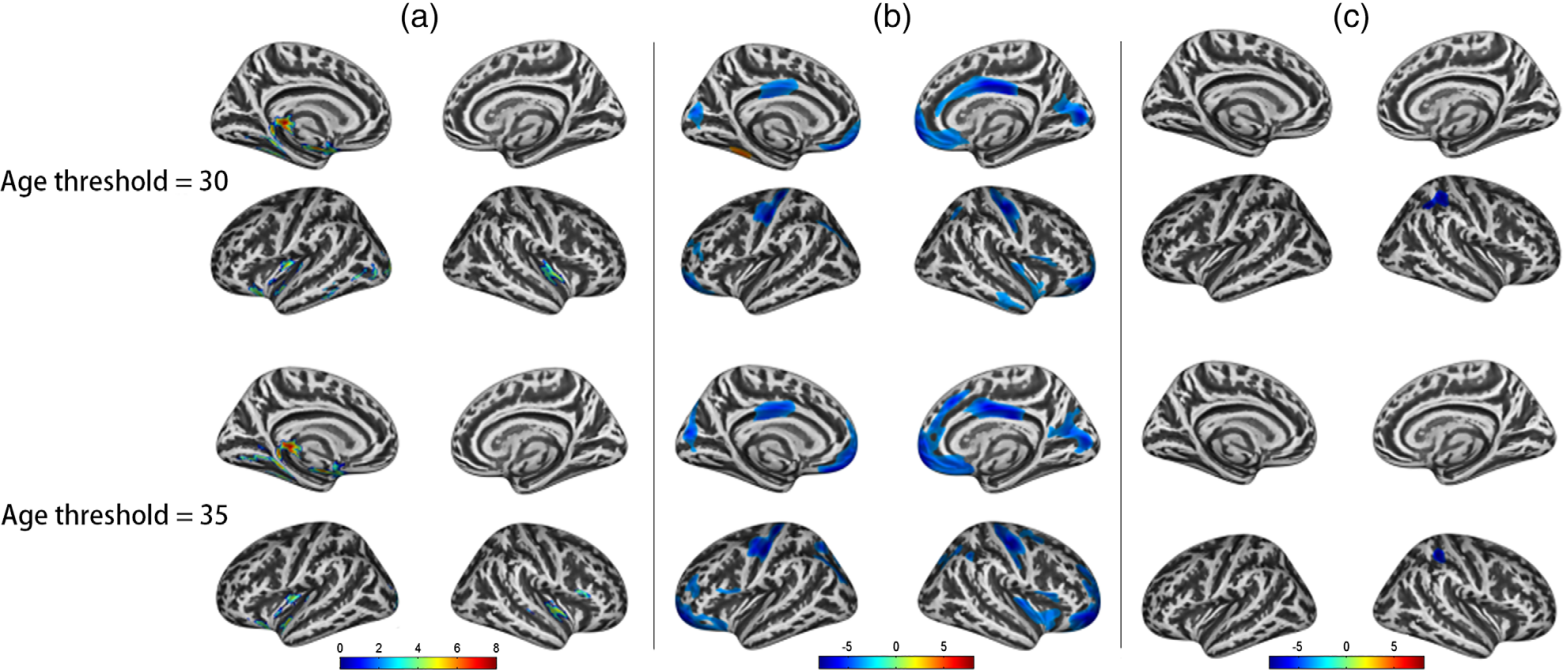
Structural differences between human immune deficiency virus (HIV) patients and healthy control (HC) at different age subgroups under different age thresholds. (a) Gray matter volumes (GMV) differences between young‐aged HIV patients and young‐aged HC; (b) cortical thickness (CTH) differences between young‐aged HIV patients and young‐aged HC; (c) GMV differences between middle‐aged HIV patients and middle‐aged HC

In most cross‐sectional studies, healthy controls are not divided into young‐aged and middle‐aged subgroups because the adult brain is thought stable during adulthood. However, neuroplasticity could also cause obvious structural alterations in matured brain (Kwok et al., 2011; Schmidt et al., 2021; Ziegler et al., 2012), therefore, age‐specific structural changes in healthy controls should not be neglected. In current study, widespread structural changes were found between young‐aged and middle‐aged healthy controls (Figure S2), demonstrating a nonuniform structural pattern in healthy controls. On the other hand, if the healthy controls were not divided into age‐specific subgroups but used as the usual way, the discovered group abnormalities looked largely different from the age‐specific group abnormalities, and the discriminative performance for HIV patients was just moderate (best accuracy = 0.83) because some sensitive information were biased by age. Additionally, there was no definite age threshold for young‐aged and middle‐aged subjects, we just selected 30 years old as threshold for the balanced samples in two age subgroups. Moreover, we wondered whether the age‐dependent structural alterations in HIV patients would conserve in another age threshold, so the age threshold was changed to 35 and the statistical analysis were repeated accordingly. The results showed the age‐dependent structural alterations in HIV patients still existed, and even the abnormality patterns were similar (Table S10, Figure 5). In addition, young‐aged and middle‐aged HIV patients also displayed distinct long‐term medication effects, and young‐aged patients were more beneficial to long‐term duration of medication. Taken together, age should not be overlooked in studying brain structural characteristics of HIV patients.

Among four structural metrics, CTH was the most sensitive to detect HIV‐related structural abnormalities no matter on whole dataset or on any age subgroups, which could also be demonstrated by the classification performances in Table S8. For every single feature, CTH achieved the highest performance, while GMV, SD, and GI were relatively low. When they were combined together, the classification performance would further improve, which was consistent with our previous study (Ma et al., 2020), implying that CTH could not replace any other three metrics and other three metrics could provide their own contributions to the HIV classification. Moreover, when detecting the long‐term medication effects, GMV was the only effective index to detect the brain structural modulation by medication, indicating that CTH was not always the optimal metric in any situation. Several studies have also reported the sensitivity of volumetric MRI even in the early stage of HIV infection (Becker et al., 2012; Benjamin et al., 2016; Wright et al., 2016). Therefore, combination of CTH and GMV may effectively detect the HIV influences on brain structure. Moreover, the latent differences between volume‐based structural metric (e.g., GMV) and surface‐based structural metric (e.g., CTH) may arise from the registration manner, and registration accuracy and reliability of volume‐based structural metric could improve once some novel deep learning‐based registration algorithms (Fan, Cao, Wang, Yap, & Shen, 2019; Fan, Cao, Yap, & Shen, 2019) are adopted.

Consistent with previous studies (Pfefferbaum et al., 2018; Underwood et al., 2017; van Zoest et al., 2017), the abnormalities in GMV/CTH were observed in HIV‐positive patients compared with HIV‐negative controls, mainly including subcortical hippocampus, thalamus, putamen and insula. Subcortical shape analysis showed that interactive effects of HIV and age existed especially in caudate, putamen, hippocampus, and thalamus (Kuhn et al., 2017). Previously, we used resting‐state fMRI to uncover that basal ganglia and frontal lobe were related to serostatus conversion through simian infected macaque model, and the longitudinal observations further pointed out that precentral gyrus and anterior cingulate cortex were also involved (Zhao et al., 2017; Zhao et al., 2019). Taken together, subcortical regions exhibit structural vulnerability to HIV infection. Furthermore, our results showed long‐term therapy alleviated the tendency of accelerating brain atrophy in HIV patients, which were consistent with previous studies that discovered early initiation of HAART might be beneficial to brain integrity (Becker et al., 2011; Sanford et al., 2017) and may partially reverse the aging progression (Boban, Kozic, Brkic, Lendak, & Thurnher, 2018). In addition, abnormalities in CTH and SD were found correlations with cognitive attention and motor functions, which were reported disruption in early HIV infection and related to nigrostriatal pathways and prefrontal–subcortical circuitry (Chang, Ernst, Leonido‐Yee, & Speck, 2000; Everall, Luthert, & Lantos, 1991; Goodkin et al., 2017).

Several limitations should be mentioned. (a) The subject number is not large enough, and a multicenter dataset will further verify the findings in the study. (b) Differences in short/long‐term medication duration remained between young‐aged and middle‐aged HIV patients, which may bias the medication effects. Further studies could be designed to use longitudinal data of both HIV patients and healthy controls to prove the results.

## 5 CONCLUSION

In this study, we firstly reported that the young adult HIV patients suffered more severe structural damages than middle‐aged HIV patients, but were more beneficial in the long‐term cART medication than middle‐aged HIV patients. Taken together, HIV‐related structural alterations are dependent on age, and future studies should control the age information in order to ascertain the HIV‐specific alterations.

## CONFLICT OF INTEREST

The authors declare that the research was conducted in the absence of any commercial or financial relationships with potential conflicts of interest.

## AUTHOR CONTRIBUTIONS

**Jing Zhao**: Design and conceptualization of the study, analysis the data, and drafting and revising the manuscript. **Zhe Ma**: Conceptualization of the study, analysis the data, and drafting and revising the manuscript. **Feng Chen**: Analysis and interpretation the data and drafting the manuscript. **Li Li**: Analysis and interpretation the data and drafting the manuscript. **Meiji Ren**: Analysis and interpretation the data and drafting the manuscript. **Aixin Li**: Analysis and interpretation the data and drafting the manuscript. **Bin Jing**: Design and conceptualization of the study, analysis the data, and revising the manuscript. **Hongjun Li**: Design and conceptualization of the study, analysis the data, and revising the manuscript.

## Supporting information


**Appendix** **S1:** Supplementary Information

## Data Availability

The data that support the findings of this study are available from the corresponding author upon reasonable request.

## References

[hbm25423-bib-0001] Ashburner, J. , Barnes, G. , Chen, C.‐C. , Daunizeau, J. , Flandin, G. , Friston, K. , … Phillips, C. (2018). SPM12 Manual. [Manual]. Retrieved from https://www.fil.ion.ucl.ac.uk/spm/

[hbm25423-bib-0002] Becker, J. T. , Maruca, V. , Kingsley, L. A. , Sanders, J. M. , Alger, J. R. , Barker, P. B. , … Multicenter, A. C. S. (2012). Factors affecting brain structure in men with HIV disease in the post‐HAART era. Neuroradiology, 54(2), 113–121. 10.1007/s00234-011-0854-2 21424708 PMC3154580

[hbm25423-bib-0003] Becker, J. T. , Sanders, J. , Madsen, S. K. , Ragin, A. , Kingsley, L. , Maruca, V. , … Multicenter, A. C. S. (2011). Subcortical brain atrophy persists even in HAART‐regulated HIV disease. Brain Imaging and Behavior, 5(2), 77–85. 10.1007/s11682-011-9113-8 21264551 PMC3082694

[hbm25423-bib-0004] Benjamin, L. A. , Corbett, E. L. , Connor, M. D. , Mzinganjira, H. , Kampondeni, S. , Choko, A. , … Solomon, T. (2016). HIV, antiretroviral treatment, hypertension, and stroke in Malawian adults: A case‐control study. Neurology, 86(4), 324–333. 10.1212/WNL.0000000000002278 26683649 PMC4776088

[hbm25423-bib-0005] Boban, J. M. , Kozic, D. B. , Brkic, S. V. , Lendak, D. F. , & Thurnher, M. M. (2018). Early introduction of cART reverses brain aging pattern in well‐controlled HIV infection: A comparative MR spectroscopy study. Frontiers in Aging Neuroscience, 10, 329. 10.3389/fnagi.2018.00329 30405398 PMC6200868

[hbm25423-bib-0006] Chang, L. , Ernst, T. , Leonido‐Yee, M. , & Speck, O. (2000). Perfusion MRI detects rCBF abnormalities in early stages of HIV‐cognitive motor complex. Neurology, 54(2), 389–396. 10.1212/wnl.54.2.389 10668700

[hbm25423-bib-0007] Chu, K. , Tran, T. , Wei, K. , Lammering, J. C. , Sondergaard, A. , Mogadam, E. , … King, K. S. (2018). Distinguishing brain impact of aging and HIV severity in chronic HIV using multiparametric MR imaging and MR spectroscopy. Open Forum Infectious Diseases, 5(10), ofy243. 10.1093/ofid/ofy243 30364402 PMC6195308

[hbm25423-bib-0008] Cole, J. H. , Caan, M. W. A. , Underwood, J. , De Francesco, D. , van Zoest, R. A. , Wit, F. , … COmorBidity in Relation to AIDS (COBRA) Collaboration . (2018). No evidence for accelerated aging‐related brain pathology in treated human immunodeficiency virus: Longitudinal neuroimaging results from the Comorbidity in Relation to AIDS (COBRA) Project. Clinical Infectious Diseases, 66(12), 1899–1909. 10.1093/cid/cix1124 29309532

[hbm25423-bib-0009] Correa, D. G. , Zimmermann, N. , Tukamoto, G. , Doring, T. , Ventura, N. , Leite, S. C. , … Gasparetto, E. L. (2016). Longitudinal assessment of subcortical gray matter volume, cortical thickness, and white matter integrity in HIV‐positive patients. Journal of Magnetic Resonance Imaging, 44(5), 1262–1269. 10.1002/jmri.25263 27079832

[hbm25423-bib-0010] Desikan, R. S. , Segonne, F. , Fischl, B. , Quinn, B. T. , Dickerson, B. C. , Blacker, D. , … Killiany, R. J. (2006). An automated labeling system for subdividing the human cerebral cortex on MRI scans into gyral based regions of interest. NeuroImage, 31(3), 968–980. 10.1016/j.neuroimage.2006.01.021 16530430

[hbm25423-bib-0011] Eggers, C. , Arendt, G. , Hahn, K. , Husstedt, I. W. , Maschke, M. , Neuen‐Jacob, E. , … German Association of Neuro‐AIDS und Neuro‐Infectiology (DGNANI) . (2017). HIV‐1‐associated neurocognitive disorder: Epidemiology, pathogenesis, diagnosis, and treatment. Journal of Neurology, 264(8), 1715–1727. 10.1007/s00415-017-8503-2 28567537 PMC5533849

[hbm25423-bib-0012] Everall, I. P. , Luthert, P. J. , & Lantos, P. L. (1991). Neuronal loss in the frontal cortex in HIV infection. Lancet, 337(8750), 1119–1121. 10.1016/0140-6736(91)92786-2 1674013

[hbm25423-bib-0013] Fan, J. , Cao, X. , Wang, Q. , Yap, P. T. , & Shen, D. (2019). Adversarial learning for mono‐ or multi‐modal registration. Medical Image Analysis, 58, 101545. 10.1016/j.media.2019.101545 31557633 PMC7455790

[hbm25423-bib-0014] Fan, J. , Cao, X. , Yap, P. T. , & Shen, D. (2019). BIRNet: Brain image registration using dual‐supervised fully convolutional networks. Medical Image Analysis, 54, 193–206. 10.1016/j.media.2019.03.006 30939419 PMC6764428

[hbm25423-bib-0015] Goodkin, K. , Miller, E. N. , Cox, C. , Reynolds, S. , Becker, J. T. , Martin, E. , … Multicenter, A. C. S. (2017). Effect of ageing on neurocognitive function by stage of HIV infection: Evidence from the Multicenter AIDS Cohort Study. Lancet HIV, 4(9), e411–e422. 10.1016/S2352-3018(17)30098-X 28716545 PMC5753579

[hbm25423-bib-0016] Guang‐Bin Huang, Q.‐Y. Z. , & Siew, C.‐K. (2006). Extreme learning machine: Theory and applications. Neurocomputing, 70, 489–501. 10.1016/j.neucom.2005.12.126

[hbm25423-bib-0017] Hakkers, C. S. , Arends, J. E. , Barth, R. E. , Du Plessis, S. , Hoepelman, A. I. , & Vink, M. (2017). Review of functional MRI in HIV: Effects of aging and medication. Journal of Neurovirology, 23(1), 20–32. 10.1007/s13365-016-0483-y 27718211 PMC5329077

[hbm25423-bib-0018] Heaton, R. K. , Clifford, D. B. , Franklin, D. R., Jr. , Woods, S. P. , Ake, C. , Vaida, F. , … HNRC Group . (2010). HIV‐associated neurocognitive disorders persist in the era of potent antiretroviral therapy: CHARTER Study. Neurology, 75(23), 2087–2096. 10.1212/WNL.0b013e318200d727 21135382 PMC2995535

[hbm25423-bib-0019] Hosek, S. G. , & Zimet, G. D. (2010). Behavioral considerations for engaging youth in HIV clinical research. Journal of Acquired Immune Deficiency Syndromes, 54(Suppl 1), S25–S30. 10.1097/QAI.0b013e3181e15c22 20571420

[hbm25423-bib-0020] Johnson, A. S. , Hall, H. I. , Hu, X. , Lansky, A. , Holtgrave, D. R. , & Mermin, J. (2014). Trends in diagnoses of HIV infection in the United States, 2002‐2011. JAMA, 312(4), 432–434. 10.1001/jama.2014.8534 25038362 PMC7249228

[hbm25423-bib-0021] Kallianpur, K. J. , Jahanshad, N. , Sailasuta, N. , Benjapornpong, K. , Chan, P. , Pothisri, M. , … SEARCH010/RV254 Study Group . (2020). Regional brain volumetric changes despite 2 years of treatment initiated during acute HIV infection. AIDS, 34(3), 415–426. 10.1097/QAD.0000000000002436 31725432 PMC6994348

[hbm25423-bib-0022] Kim‐Chang, J. J. , Donovan, K. , Loop, M. S. , Hong, S. , Fischer, B. , Venturi, G. , … Adolescent Medicine Trials Network for HIV/AIDS Interventions . (2019). Higher soluble CD14 levels are associated with lower visuospatial memory performance in youth with HIV. AIDS, 33(15), 2363–2374. 10.1097/QAD.0000000000002371 31764101 PMC6905124

[hbm25423-bib-0023] Kooij, K. W. , Wit, F. W. , Schouten, J. , van der Valk, M. , Godfried, M. H. , Stolte, I. G. , … AGEhIV Cohort Study Group . (2016). HIV infection is independently associated with frailty in middle‐aged HIV type 1‐infected individuals compared with similar but uninfected controls. AIDS, 30(2), 241–250. 10.1097/QAD.0000000000000910 26684821

[hbm25423-bib-0024] Kuhn, T. , Kaufmann, T. , Doan, N. T. , Westlye, L. T. , Jones, J. , Nunez, R. A. , … Thames, A. D. (2018). An augmented aging process in brain white matter in HIV. Human Brain Mapping, 39(6), 2532–2540. 10.1002/hbm.24019 29488278 PMC5951745

[hbm25423-bib-0025] Kuhn, T. , Schonfeld, D. , Sayegh, P. , Arentoft, A. , Jones, J. D. , Hinkin, C. H. , … Thames, A. D. (2017). The effects of HIV and aging on subcortical shape alterations: A 3D morphometric study. Human Brain Mapping, 38(2), 1025–1037. 10.1002/hbm.23436 27778407 PMC5225033

[hbm25423-bib-0026] Kwok, V. , Niu, Z. , Kay, P. , Zhou, K. , Mo, L. , Jin, Z. , … Tan, L. H. (2011). Learning new color names produces rapid increase in gray matter in the intact adult human cortex. Proceedings of the National Academy of Sciences of the United States of America, 108(16), 6686–6688. 10.1073/pnas.1103217108 21464316 PMC3081005

[hbm25423-bib-0027] Lovden, M. , Wenger, E. , Martensson, J. , Lindenberger, U. , & Backman, L. (2013). Structural brain plasticity in adult learning and development. Neuroscience and Biobehavioral Reviews, 37(9 Pt B), 2296–2310. 10.1016/j.neubiorev.2013.02.014 23458777

[hbm25423-bib-0028] Ma, Z. , Jing, B. , Li, Y. , Yan, H. , Li, Z. , Ma, X. , … Alzheimer's Disease Neuroimaging Initiative . (2020). Identifying mild cognitive impairment with random forest by integrating multiple MRI morphological metrics. Journal of Alzheimer's Disease, 73(3), 991–1002. 10.3233/JAD-190715 31884464

[hbm25423-bib-0029] MacDuffie, K. E. , Brown, G. G. , McKenna, B. S. , Liu, T. T. , Meloy, M. J. , Tawa, B. , … Igor Grant . (2018). Effects of HIV infection, methamphetamine dependence and age on cortical thickness, area and volume. NeuroImage: Clinical, 20, 1044–1052. 10.1016/j.nicl.2018.09.034 30342393 PMC6197439

[hbm25423-bib-0030] Pfefferbaum, A. , Zahr, N. M. , Sassoon, S. A. , Kwon, D. , Pohl, K. M. , & Sullivan, E. V. (2018). Accelerated and premature aging characterizing regional cortical volume loss in human immunodeficiency virus infection: Contributions from alcohol, substance use, and hepatitis C coinfection. Biological Psychiatry: Cognitive Neuroscience and Neuroimaging, 3(10), 844–859. 10.1016/j.bpsc.2018.06.006 30093343 PMC6508083

[hbm25423-bib-0031] Qiao, Y. C. , Xu, Y. , Jiang, D. X. , Wang, X. , Wang, F. , Yang, J. , & Wei, Y. S. (2019). Epidemiological analyses of regional and age differences of HIV/AIDS prevalence in China, 2004‐2016. International Journal of Infectious Diseases, 81, 215–220. 10.1016/j.ijid.2019.02.016 30797071

[hbm25423-bib-0032] Ragin, A. B. , Du, H. , Ochs, R. , Wu, Y. , Sammet, C. L. , Shoukry, A. , & Epstein, L. G. (2012). Structural brain alterations can be detected early in HIV infection. Neurology, 79(24), 2328–2334. 10.1212/WNL.0b013e318278b5b4 23197750 PMC3578377

[hbm25423-bib-0033] Sanford, R. , Ances, B. M. , Meyerhoff, D. J. , Price, R. W. , Fuchs, D. , Zetterberg, H. , … Collins, D. L. (2018). Longitudinal trajectories of brain volume and cortical thickness in treated and untreated primary human immunodeficiency virus infection. Clinical Infectious Diseases, 67(11), 1697–1704. 10.1093/cid/ciy362 29697762 PMC6233681

[hbm25423-bib-0034] Sanford, R. , Fellows, L. K. , Ances, B. M. , & Collins, D. L. (2018). Association of brain structure changes and cognitive function with combination antiretroviral therapy in HIV‐positive individuals. JAMA Neurology, 75(1), 72–79. 10.1001/jamaneurol.2017.3036 29131878 PMC5833491

[hbm25423-bib-0035] Sanford, R. , Fernandez Cruz, A. L. , Scott, S. C. , Mayo, N. E. , Fellows, L. K. , Ances, B. M. , & Collins, D. L. (2017). Regionally specific brain volumetric and cortical thickness changes in HIV‐infected patients in the HAART era. Journal of Acquired Immune Deficiency Syndromes, 74(5), 563–570. 10.1097/QAI.0000000000001294 28129254 PMC5340610

[hbm25423-bib-0036] Saylor, D. , Dickens, A. M. , Sacktor, N. , Haughey, N. , Slusher, B. , Pletnikov, M. , … McArthur, J. C. (2016). HIV‐associated neurocognitive disorder—Pathogenesis and prospects for treatment. Nature Reviews. Neurology, 12(5), 309. 10.1038/nrneurol.2016.53 PMC5842923 27080521

[hbm25423-bib-0037] Schmidt, S. , Gull, S. , Herrmann, K. H. , Boehme, M. , Irintchev, A. , Urbach, A. , … Witte, O. W. (2021). Experience‐dependent structural plasticity in the adult brain: How the learning brain grows. NeuroImage, 225, 117502. 10.1016/j.neuroimage.2020.117502 33164876

[hbm25423-bib-0038] Schouten, J. , Wit, F. W. , Stolte, I. G. , Kootstra, N. A. , van der Valk, M. , Geerlings, S. E. , … AGEhIV Cohort Study Group . (2014). Cross‐sectional comparison of the prevalence of age‐associated comorbidities and their risk factors between HIV‐infected and uninfected individuals: The AGEhIV cohort study. Clinical Infectious Diseases, 59(12), 1787–1797. 10.1093/cid/ciu701 25182245

[hbm25423-bib-0039] Sui, J. , Li, X. , Bell, R. P. , Towe, S. L. , Gadde, S. , Chen, N. K. , & Meade, C. S. (2020). Structural and functional brain abnormalities in HIV disease revealed by multimodal MRI fusion: Association with cognitive function. Clinical Infectious Diseases. 10.1093/cid/ciaa1415 Online ahead of print. PMC8492163 32948879

[hbm25423-bib-0040] Underwood, J. , Cole, J. H. , Caan, M. , de Francesco, D. , Leech, R. , van Zoest, R. A. , … for the Comorbidity in Relation to AIDS (COBRA) Collaboration . (2017). Gray and white matter abnormalities in treated human immunodeficiency virus disease and their relationship to cognitive function. Clinical Infectious Diseases, 65(3), 422–432. 10.1093/cid/cix301 28387814 PMC5850629

[hbm25423-bib-0041] van Zoest, R. A. , Underwood, J. , de Francesco, D. , Sabin, C. A. , Cole, J. H. , Wit, F. W. , … Comorbidity in Relation to AIDS (COBRA) Collaboration . (2017). Structural brain abnormalities in successfully treated HIV infection: Associations with Disease and cerebrospinal fluid biomarkers. The Journal of Infectious Diseases, 217(1), 69–81. 10.1093/infdis/jix553 29069436

[hbm25423-bib-0042] Wright, P. W. , Pyakurel, A. , Vaida, F. F. , Price, R. W. , Lee, E. , Peterson, J. , … Ances, B. M. (2016). Putamen volume and its clinical and neurological correlates in primary HIV infection. AIDS, 30(11), 1789–1794. 10.1097/QAD.0000000000001103 27045376 PMC4925211

[hbm25423-bib-0043] Zhang, Y. , Liu, Y. , Chao, H. C. , Zhang, Z. , & Zhang, Z. (2018). Classification of incomplete data based on evidence theory and an extreme learning machine in wireless sensor networks. Sensors (Basel), 18(4), 1046. 10.3390/s18041046 PMC5948797 29601552

[hbm25423-bib-0044] Zhao, J. , Chen, F. , Ren, M. , Li, L. , Li, A. , Jing, B. , & Li, H. (2019). Low‐frequency fluctuation characteristics in rhesus macaques with SIV infection: A resting‐state fMRI study. Journal of Neurovirology, 25(2), 141–149. 10.1007/s13365-018-0694-5 30478797

[hbm25423-bib-0045] Zhao, J. , Jing, B. , Chen, F. , Liu, J. , Wang, Y. , & Li, H. (2017). Altered regional homogeneity of brain spontaneous signals in SIV infected rhesus macaque model. Magnetic Resonance Imaging, 37, 56–61. 10.1016/j.mri.2016.10.019 27989909

[hbm25423-bib-0046] Ziegler, G. , Dahnke, R. , Jancke, L. , Yotter, R. A. , May, A. , & Gaser, C. (2012). Brain structural trajectories over the adult lifespan. Human Brain Mapping, 33(10), 2377–2389. 10.1002/hbm.21374 21898677 PMC6870331

