# Supplementary material for: Human immune deficiency virus‐related structural alterations in the brain are dependent on age
Source: Hum Brain Mapp. 2021 Mar 23;42(10):3131–40. doi: 10.1002/hbm.25423 (PMC8193536; doi:10.1002/hbm.25423)
Supplement: Supplementary file 1 — Appendix S1: Supplementary Information [file HBM-42-3131-s001.docx]

**
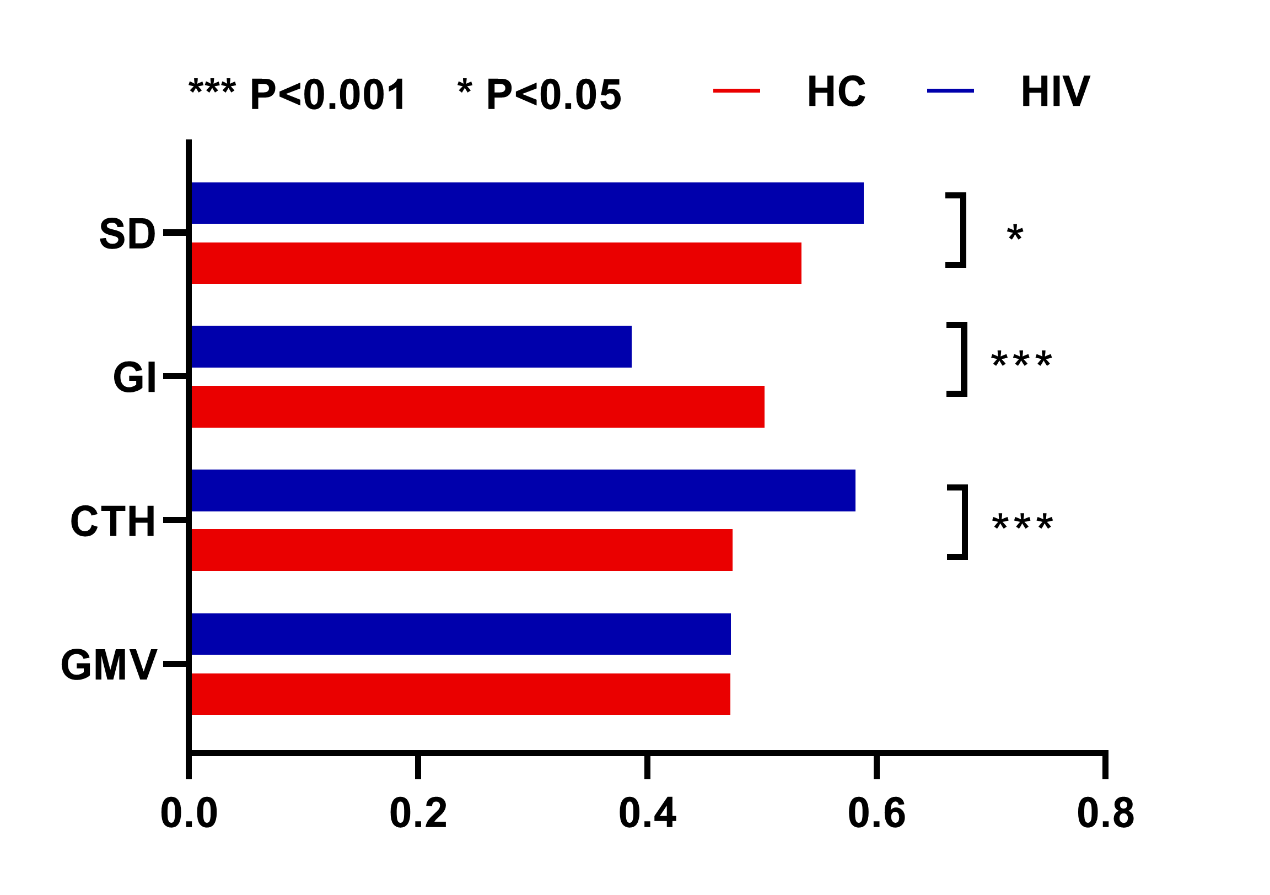
**

Figure S1. Global mean differences for each structural metric between HC and HIV on whole dataset (The horizontal-axis represents the normalized value of each feature).


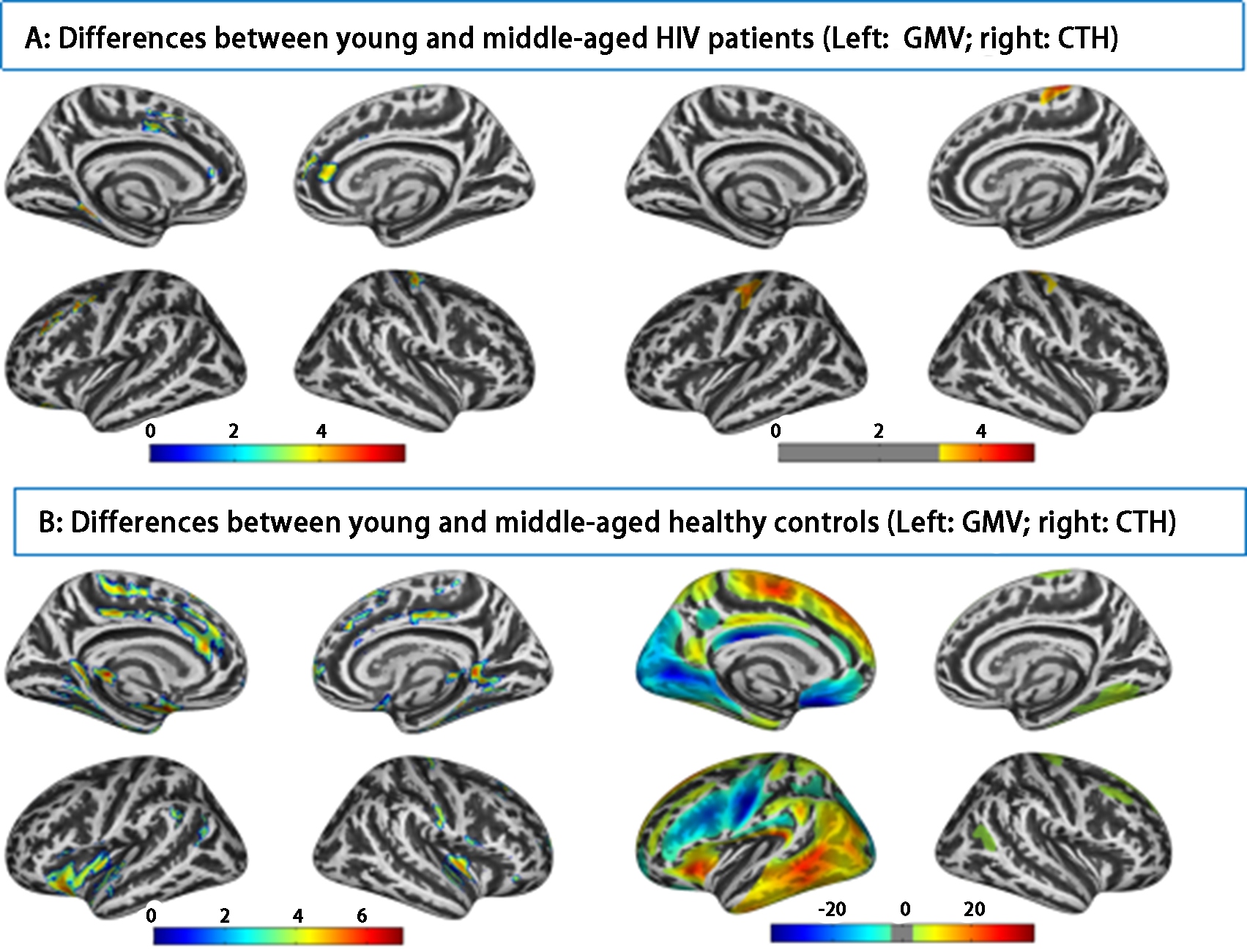


Figure S2. Significant differences on GMV and CTH between young and middle-aged HIV/HC subjects.

Figure S3. Age-dependent differences between HIV and HC groups on GI and SD respectively (HC>HIV).


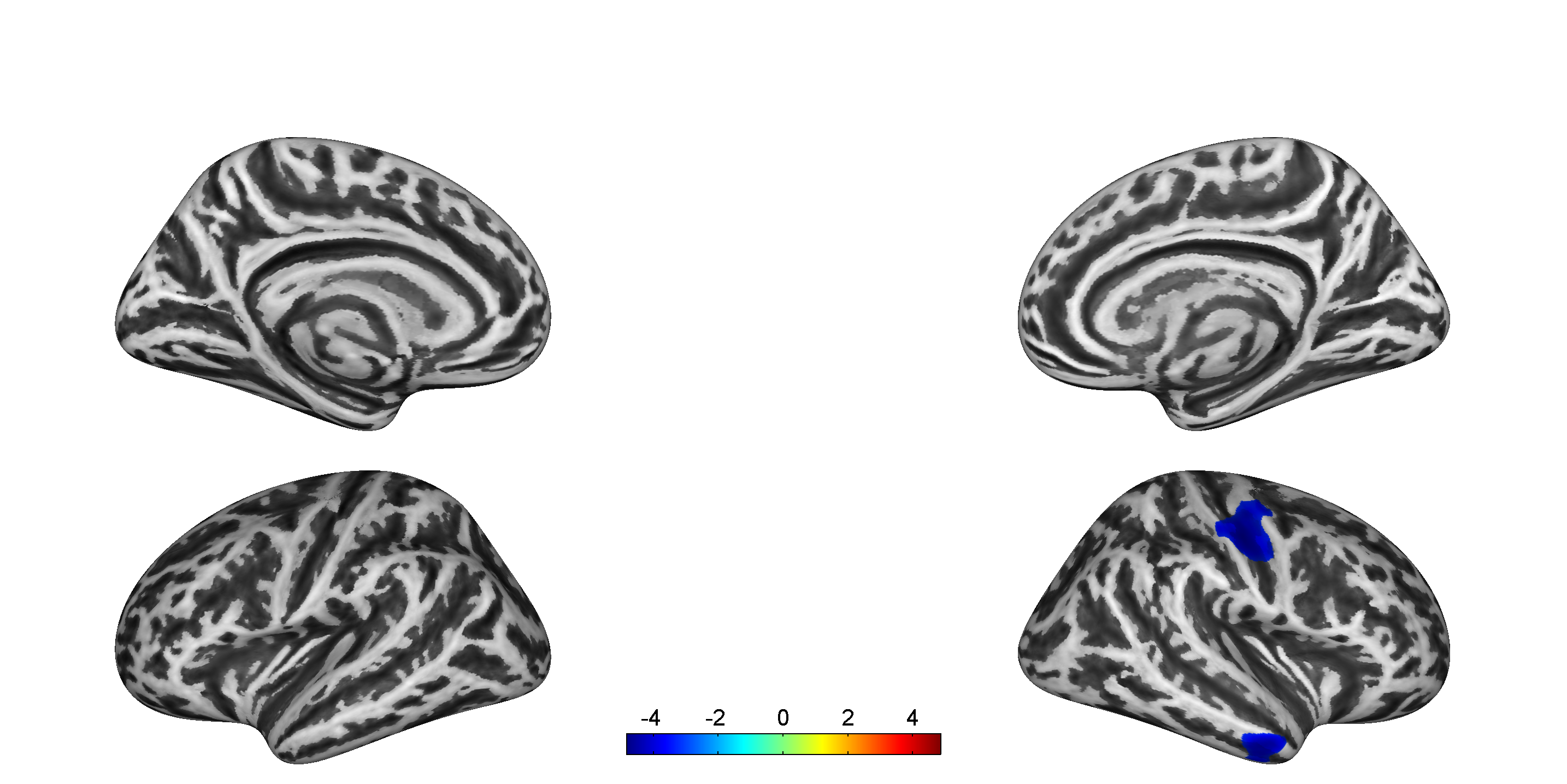

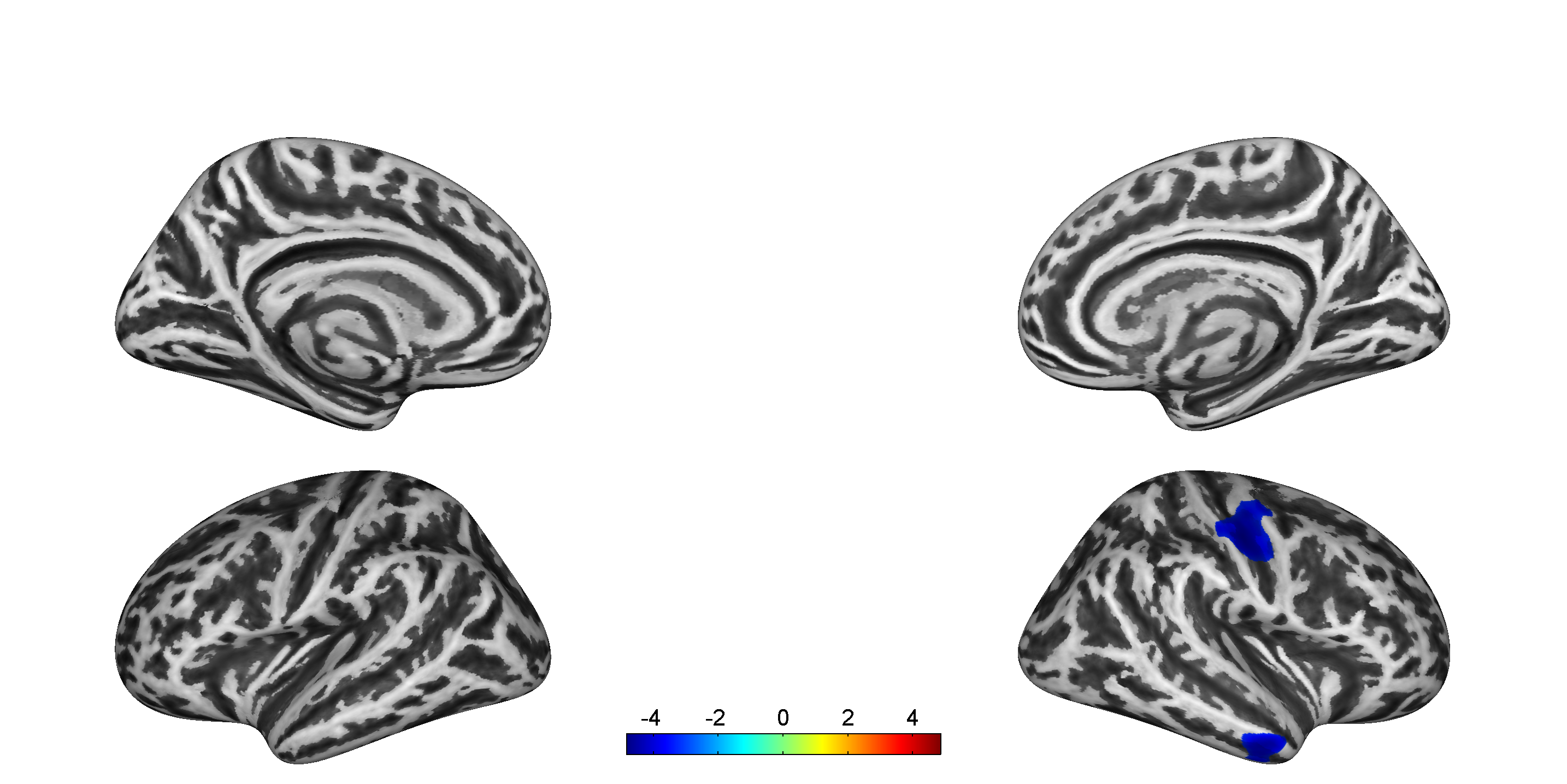

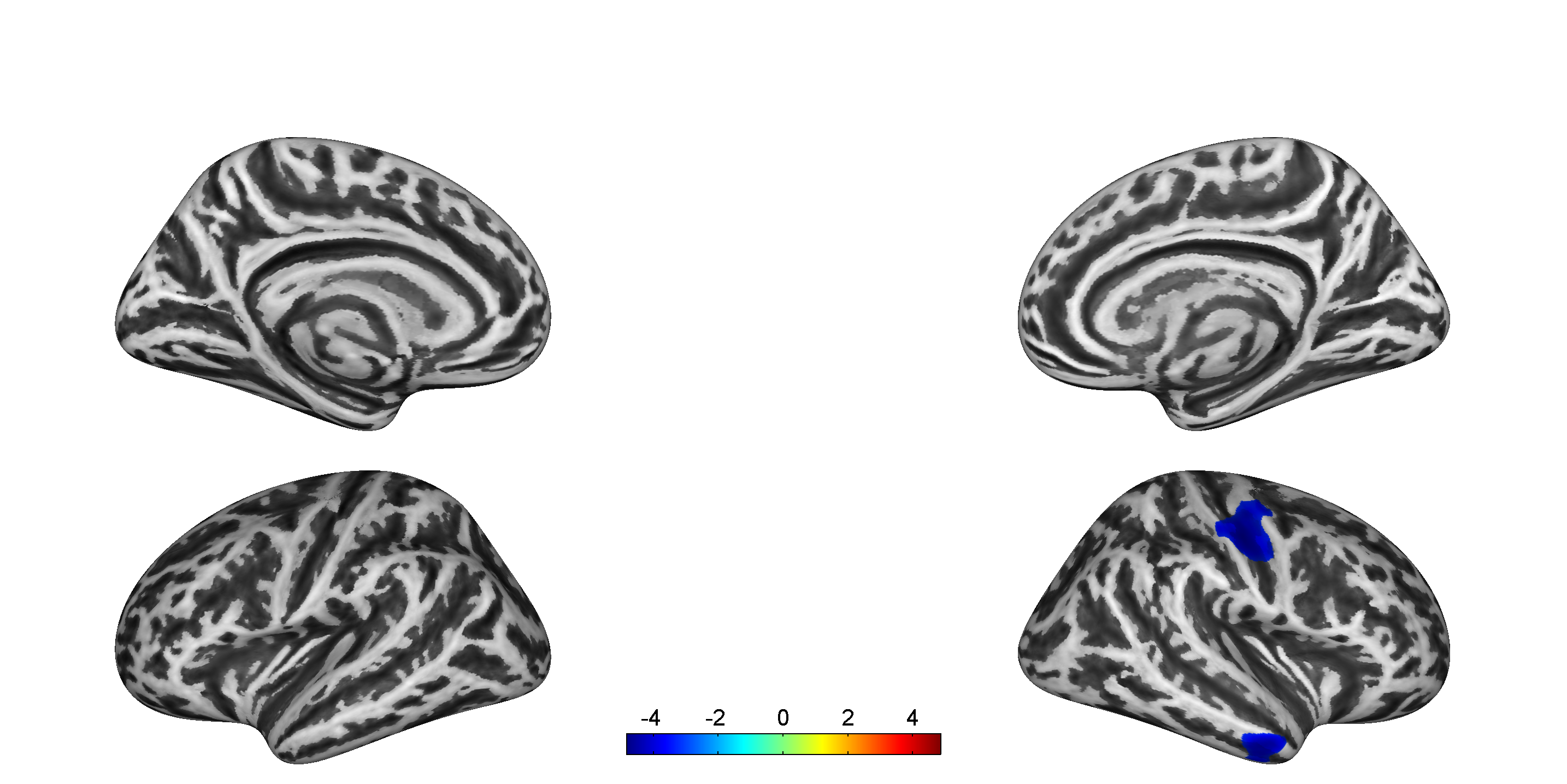

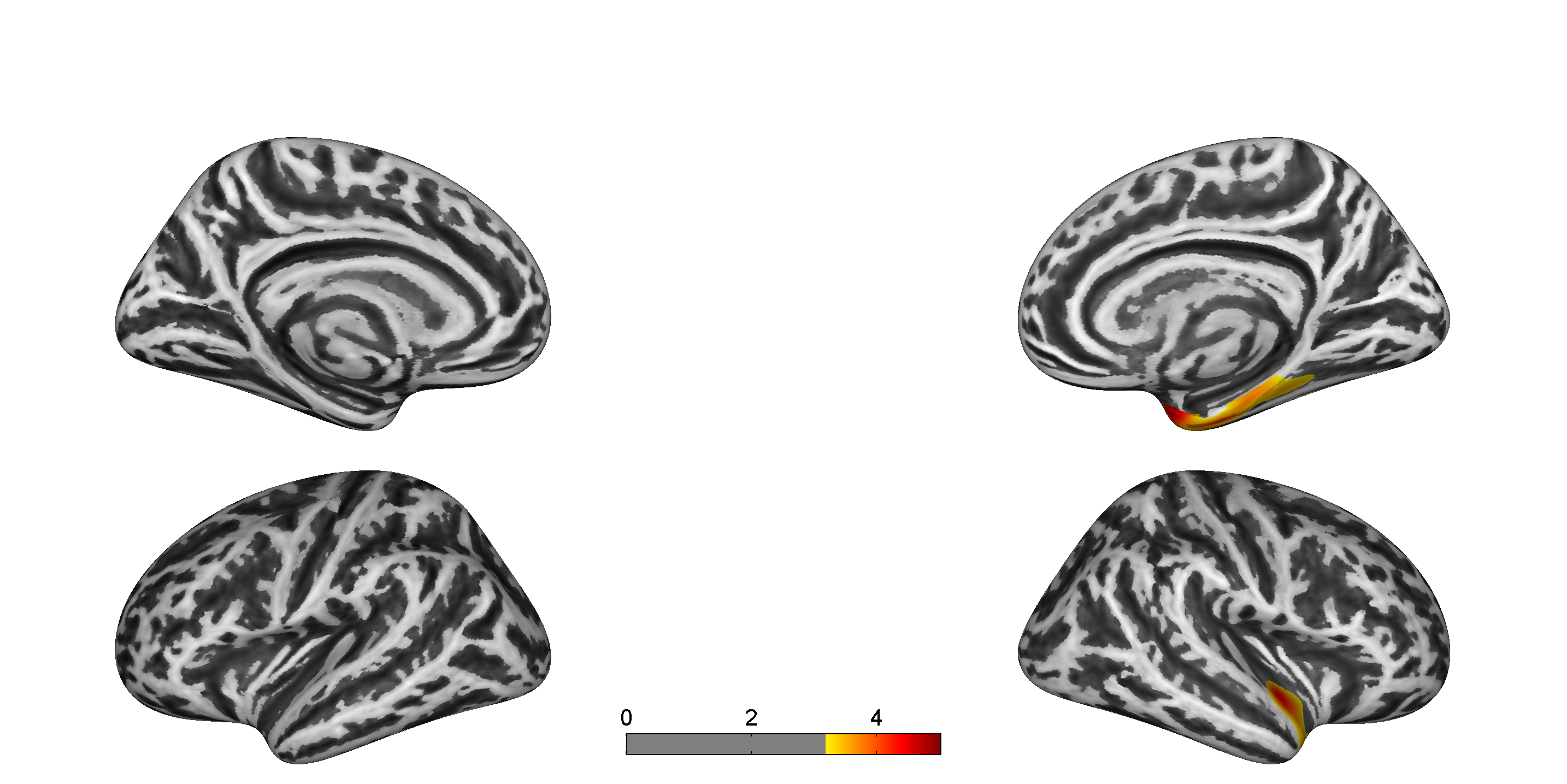

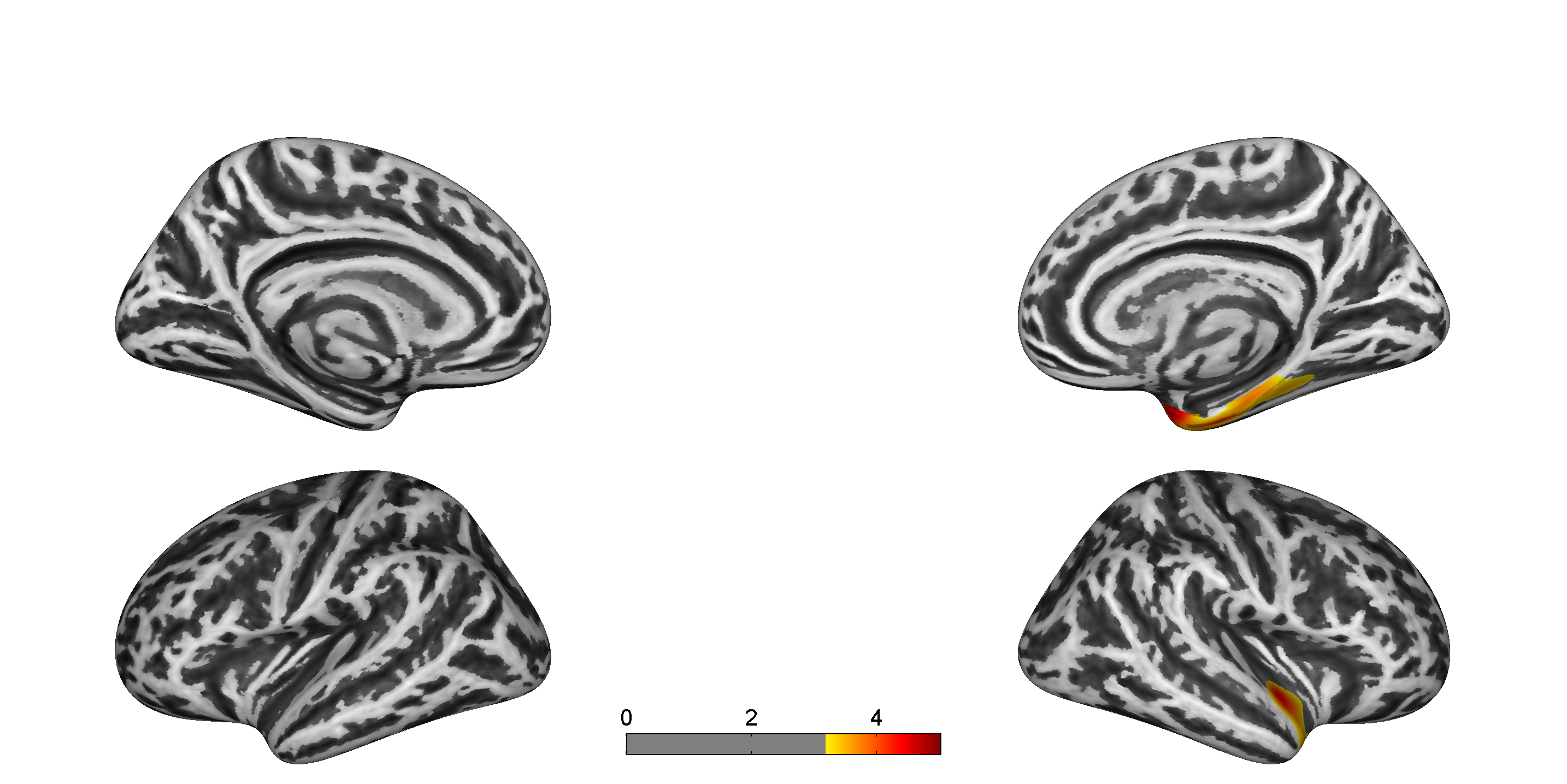

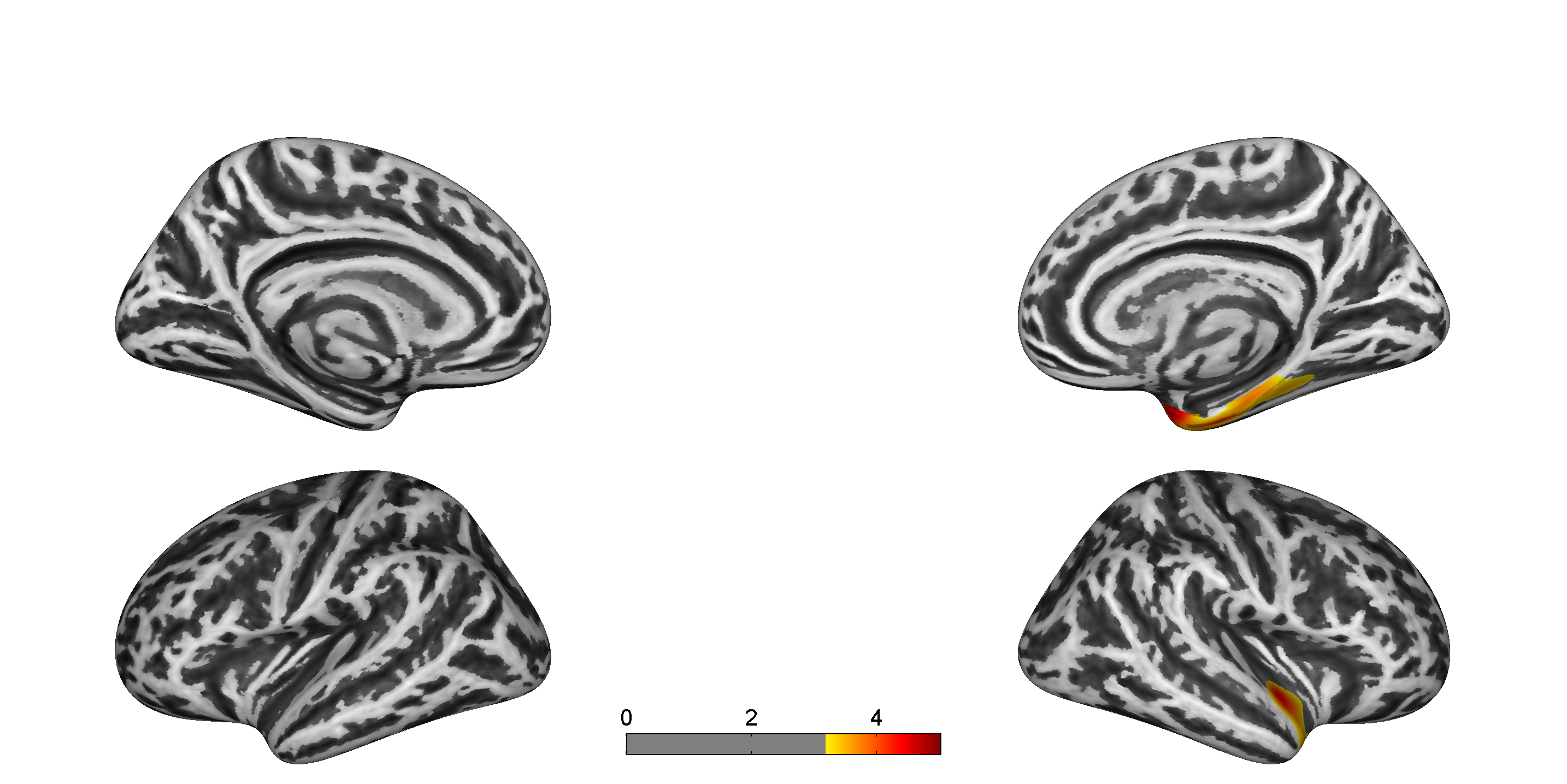


**GI alterations in Young-aged groups**

**SD alterations in Middle-aged groups**


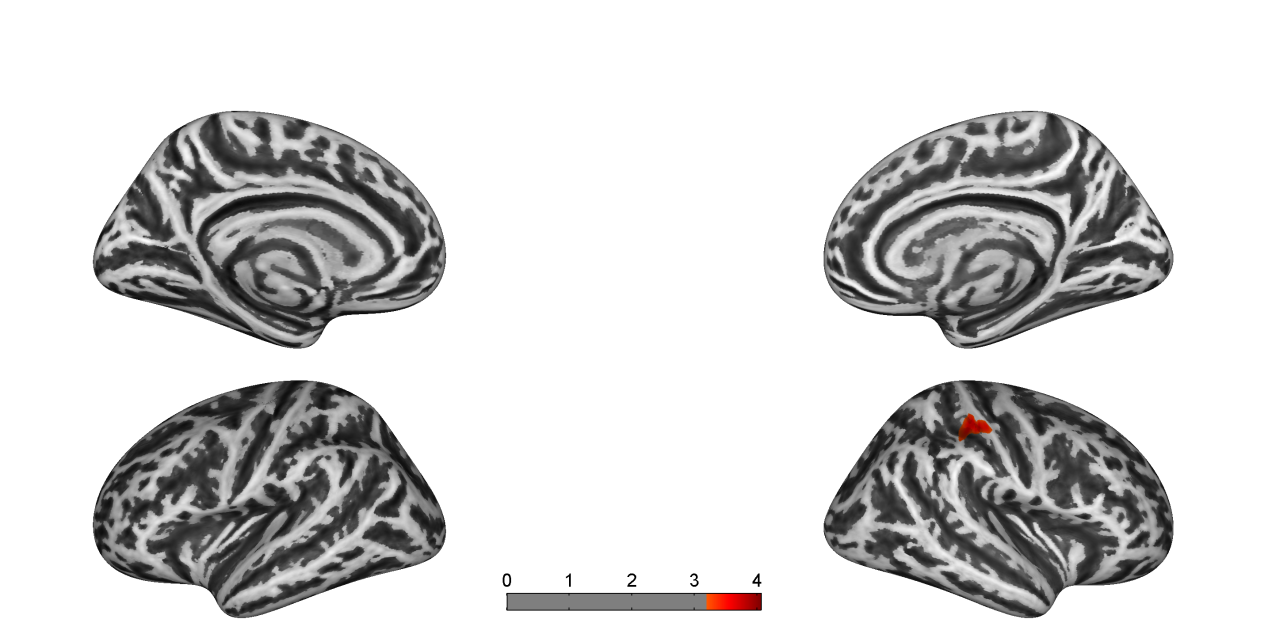

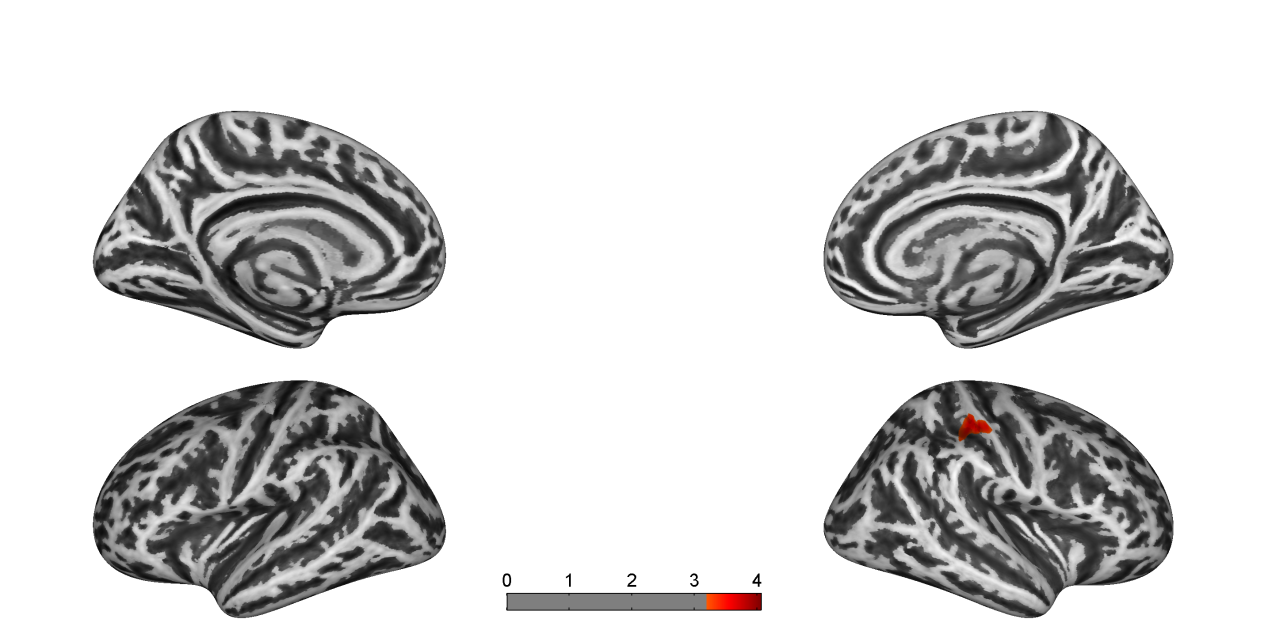

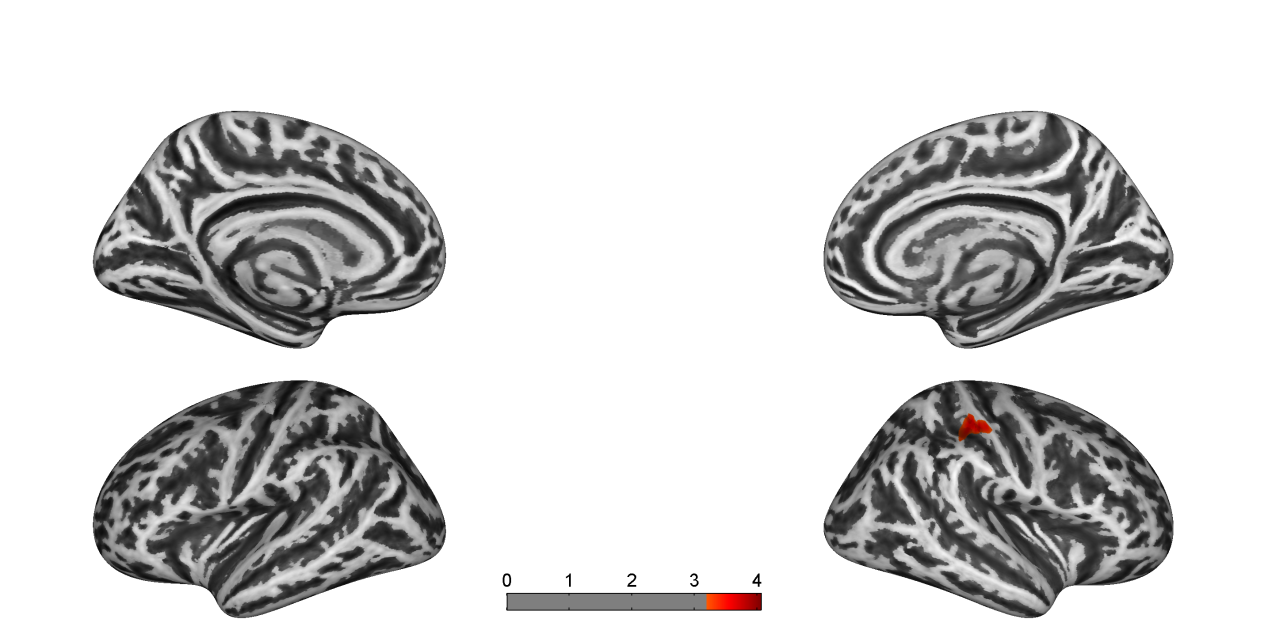


Figure S4. Differences on SD between young-aged and middle-aged patients with HIV infection (young > middle).

Table S1. Correlations between demographic information and age on whole HIV patients

|  | Age | |
| --- | --- | --- |
|  | R value | P value |
| Language fluency | 0.16 | 0.243 |
| **Attention** | **0.37** | **0.004** |
| Executive function | 0.01 | 0.933 |
| Memory | 0.06 | 0.671 |
| Speed of information processing | 0.22 | 0.102 |
| Motor function | 0.01 | 0.924 |
| CD4 | 0.09 | 0.489 |
| CD4/CD8 | 0.11 | 0.415 |

Table S2. Differences in the cognitive domain between young-aged and middle-aged HIV patients

|  | Young-aged | Middle-aged | P value |
| --- | --- | --- | --- |
| Language fluency | 43.6 (8.9) | 47.6 (9.8) | 0.053 |
| **Attention** | **38.7 (7.0)** | **45.2(8.3)** | **0.001*** |
| Executive function | 54.8 (10.5) | 56.7 (8.8) | 0.431 |
| Memory | 41.7 (7.8) | 45.2 (7.7) | 0.059 |
| Speed of information processing | 42.9 (9.8) | 46.6 (8.2) | 0.096 |
| Motor function | 44.7 (8.5) | 46.8 (11.2) | 0.384 |

*Significant difference. if data satisfy the standard normal distribution, two sample T test is used, else Mann-Whitney U test is adopted.

Table S3. Differences between young-aged HIV and controls (see Table S9 for the full names of brain regions).

| Contrast | Feature | Brain regions | Cluster size | T value |
| --- | --- | --- | --- | --- |
| HC>HIV | GMV | L, PHIP/FUS | 373 | 4.3 |
|  |  | L, ITG/MTG | 437 | 4.5 |
|  |  | L, Tha/FUS/HIP/LING | 2530 | 7.5 |
|  |  | L, INS/Olf | 776 | 4.9 |
|  |  | R, INS/ST | 938 | 5.4 |
|  |  | L, ST | 344 | 5.0 |
|  |  | L, PUT/PALL/AMYG | 3773 | 7.1 |
|  |  | R, PUT/PALL/INS | 3229 | 6.6 |
|  |  | L, MO/MTG/IO | 376 | 4.7 |
|  |  | L, INS/HES | 646 | 5.7 |
|  |  | L, MTG | 443 | 4.6 |
|  | CTH | L, PREC/PC | 4407 | -7.4 |
|  |  | L, MF | 3986 | -6.0 |
|  |  | L, PCC | 1404 | -4.8 |
|  |  | L, IP | 1350 | -4.5 |
|  |  | L, cuneus | 939 | -4.7 |
|  |  | L, FUS | 818 | **4.6** |
|  |  | R, MF | 6530 | -6.6 |
|  |  | R, PREC | 3906 | -6.8 |
|  |  | R, PCC | 2570 | -6.2 |
|  |  | R, INS | 1724 | -4.3 |
|  |  | R, MTG/ITG | 924 | -3.8 |
|  |  | R, PO | 766 | -4.2 |
|  |  | R, SP/IP | 614 | -4.3 |
|  | GI | R, ST/PHIP/FUS | 4593 | 4.6 |

L, left; R, right; B, bilateral;

Table S4. Differences between middle-aged HIV and controls.

| Contrast | Feature | Brain regions | Cluster size | T value |
| --- | --- | --- | --- | --- |
| HIV > HC | CTH | R, SP/IP | 2249 | 4.8 |
|  | SD | R, PREC/PC | 2776 | 4.1 |
|  |  | R, MTG/ITG | 807 | 4.4 |

Table S5. Differences between young-aged and middle-aged HIV patients.

|  | Feature | Brain regions | Cluster size | T value |
| --- | --- | --- | --- | --- |
| young >  middle | GMV | L, SF/IF/MF | 536 | 4.5 |
|  |  | P, PUT/PALL | 1044 | 4.7 |
|  |  | R, AC | 716 | 4.2 |
|  |  | L, MF | 859 | 5.6 |
|  |  | B, PC | 2204 | 5.2 |
|  | CTH | L, PREC/PC | 1934 | 3.9 |
|  |  | R, PREC | 2107 | 4.6 |
|  | SD | R, PC/SMG | 1027 | 3.7 |

Table S6. Differences between young-aged and middle-aged controls.

| Contrast | Feature | Brain regions | Cluster size | T value |
| --- | --- | --- | --- | --- |
| young > middle | GMV | L, MTG/ITG | 638 | 4.3 |
|  |  | L, Tha/CAL | 1178 | 6.8 |
|  |  | L, Caudate | 819 | 4.1 |
|  |  | L, AC | 1538 | 5.4 |
|  |  | L, MC | 2701 | 5.4 |
|  |  | R, PC/SMG | 1071 | 5.5 |
|  |  | R, AMYG/HIP | 626 | 6.2 |
|  |  | B, PUT/PALL | 8749 | 9.2 |
|  |  | B, SF | 1042 | 6.0 |
|  |  | B, PC | 1085 | 4.8 |
|  |  | B, LING | 2186 | 5.8 |
|  |  | B, FUS/PHIP | 1411 | 5.9 |
|  | CTH | L, IP/IT/MT | 24493 | 26.7 |
|  |  | L, SF/ParaC | 14514 | 25.7 |
|  |  | L, INS | 6262 | 26.4 |
|  |  | L, SP | 7025 | 12.4 |
|  |  | L, PCC | 1401 | 12.1 |
|  |  | L, PC | 1305 | 10.3 |
|  |  | L, PREC | 1974 | 10.4 |
|  |  | L, LING/SP | 16295 | -29.4 |
|  |  | L, PREC/PC | 12910 | -31.9 |
|  |  | L, MF | 5632 | -34.5 |
|  |  | L, SP/IP | 2093 | -6.9 |
|  |  | L, PCC | 1583 | -33.0 |
|  |  | L, SMG/PC | 1451 | -10.2 |
|  |  | R, FUS/LING | 3364 | 6.6 |
|  |  | R, PREC/ParaC | 2432 | 5.6 |
|  |  | R, MF | 1025 | 5.5 |
|  |  | R, IP/MT | 726 | 4.1 |
|  |  | R, FUS/IT | 485 | 4.6 |

Table S7. Brain alterations between groups divided by medication duration and age.

| Contrast | Feature | Brain regions | Cluster size | T value |
| --- | --- | --- | --- | --- |
| Young > Middle  (Short-term) | GMV | L, MF | 526 | 5.0 |
| Young > Middle  (Long-term) | GMV | L, SF/AC | 1027 | 5.5 |
|  |  | L, IP | 482 | 5.8 |
|  | CTH | B, PREC/PC | 6656 | 5.9 |
| Short-term > Long-term  (Young) | GMV | L, SP/PC | 295 | -4.6 |
| Short-term> Long-term  (Middle) | GMV | L, AC | 381 | 5.0 |
|  |  | L, PREC/PC | 787 | 5.0 |

Table S8. Results of SVM classification between groups using different features.

| Type | Feature | Accuracy | Sensitivity | Specificity | Number of features |
| --- | --- | --- | --- | --- | --- |
| C1 | GMV | 0.67 | 0.75 | 0.57 | 7 |
|  | CTH | 0.72 | 0.66 | 0.78 | 18 |
|  | GI | 0.65 | 0.69 | 0.62 | 3 |
|  | SD | 0.57 | 0.61 | 0.57 | 2 |
|  | All features | 0.80 | 0.81 | 0.79 | 30 |
|  | All features+RFE | 0.83 | 0.83 | 0.82 | 21 |
| C2 | GMV | 0.73 | 0.81 | 0.73 | 12 |
|  | CTH | 0.86 | 0.94 | 0.79 | 15 |
|  | GI | 0.59 | 0.55 | 0.62 | 1 |
|  | All features | 0.90 | 0.99 | 0.81 | 28 |
|  | All features+RFE | 0.91 | 0.98 | 0.82 | 9 |
| C3 | CTH | 0.59 | 0.58 | 0.62 | 1 |
|  | SD | 0.65 | 0.63 | 0.72 | 2 |
|  | All features | 0.75 | 0.70 | 0.82 | 3 |

Table S9. Names of brain regions and corresponding abbreviations.

| Brain regions | Abbreviations |  | Brain regions | Abbreviations |
| --- | --- | --- | --- | --- |
| Putamen | PUT |  | Middle occipital gyrus | MO |
| Amygdala | AMYG |  | Middle temporal gyrus | MTG |
| Anterior Cingulum | AC |  | Olfactory | Olf |
| Calcarine | CAL |  | Pallidum | PALL |
| Fusiform | FUS |  | Paracentral | ParaC |
| Heschl | HES |  | Parahippocampal gyrus | PHIP |
| Hippocampus | HIP |  | Pars opercularis | PO |
| Inferior frontal gyrus | IF |  | Postcentral | PC |
| Inferior occipital gyrus | IO |  | Posterior Cingulum | PCC |
| Inferior parietal gyrus | IP |  | Precentral | PREC |
| Inferior temporal gyrus | ITG |  | Precuneus | PreC |
| Insula | INS |  | Superior frontal gyrus | SF |
| Lingual | LING |  | Superior parietal gyrus | SP |
| Middle Cingulum | MC |  | Superior temporal gyrus | ST |
| Middle frontal gyrus | MF |  | Supramarginal gyrus | SMG |
| Thalamus | Tha |  |  |  |

Table S10. Number of subjects in different age subgroups if age threshold is set to 35.

|  |  | Age range | |  |
| --- | --- | --- | --- | --- |
|  |  | 20-34 | 35-50 | Total |
| Subjects | HIV | 58 | 25 | 83 |
|  | HC | 59 | 24 | 83 |
|  | Total | 117 | 49 | 166 |
